# New colours for old in the blue-cheese fungus *Penicillium roqueforti*

**DOI:** 10.1038/s41538-023-00244-9

**Published:** 2024-01-08

**Authors:** Matthew M. Cleere, Michaela Novodvorska, Elena Geib, Jack Whittaker, Heather Dalton, Nadhira Salih, Sarah Hewitt, Matthew Kokolski, Matthias Brock, Paul S. Dyer

**Affiliations:** 1 School of Life Sciences, University of Nottingham, Nottingham, NG7 2RD United Kingdom; 2 PhD Program in Biology, The Graduate Center; Structural Biology Initiative, CUNY Advanced Science Research Center, New York, NY10031 USA; 3 Department of Biology, College of Education, University of Sulaimani, Sulaymaniyah, Iraq

**Keywords:** Fungal genetics, Fungal biology

## Abstract

*Penicillium roqueforti* is used worldwide in the production of blue-veined cheese. The blue-green colour derives from pigmented spores formed by fungal growth. Using a combination of bioinformatics, targeted gene deletions, and heterologous gene expression we discovered that pigment formation was due to a DHN-melanin biosynthesis pathway. Systematic deletion of pathway genes altered the arising spore colour, yielding white to yellow-green to red-pink-brown phenotypes, demonstrating the potential to generate new coloured strains. There was no consistent impact on mycophenolic acid production as a result of pathway interruption although levels of roquefortine C were altered in some deletants. Importantly, levels of methyl-ketones associated with blue-cheese flavour were not impacted. UV-induced colour mutants, allowed in food production, were then generated. A range of colours were obtained and certain phenotypes were successfully mapped to pathway gene mutations. Selected colour mutants were subsequently used in cheese production and generated expected new colourations with no elevated mycotoxins, offering the exciting prospect of use in future cheese manufacture.

## Introduction

The production of several food products relies on the essential activity of food-associated microbes. The filamentous fungi *Penicillium roqueforti* and *Penicillium camemberti* are well known for their use in the production of interior and exterior mould-ripened cheeses, respectively, in which fungal inoculum is added as a starter culture during cheese production. In the case of *P. roqueforti*, growth of the fungus leads to the production of volatile and non-volatile flavour components and changes in cheese texture due to the metabolic action of the species, as well as the characteristic blue-green veined appearance of cheeses arising from fungal sporulation^1,2^.

These fungi, as well as other microbes used in food production, have in common that they show signs of domestication, by which an environmental microorganism has been selected and become adapted to a human-provided niche accompanied by the loss of unwanted and gain of favourable properties^3–5^. Regarding *P. roqueforti* there is evidence of adaptive divergence, with the presence of wild populations found in environmental locations such as on silage and lumbar, but with separate differentiated populations found associated with certain types of cheese^2,4,6,7^. At least two such cheese populations have been detected, which show signs of domestication. These include attributes such as greater spore production on bread (traditionally used for production of fungal inoculum), high lipolytic activity and salt tolerance, adaptation to lactose utilisation and growth in an acidic environment, and successful competition with fermentative bacteria^1,4^. These traits are correlated partly with the presence of genomic regions possibly originating from horizontal gene transfer^2,8^. Advancements in the possible exploitation and domestication of *P. roqueforti* came when a sexual cycle was discovered, opening the possibility of using sexual breeding to generate ascospore offspring with new flavour profiles and other beneficial characteristics for cheese production^9–11^. The subsequent publication of the *P. roqueforti* genome has led to further opportunities for the study of the ‘blue-cheese fungus’^8^.

Considerable diversity between strains of *P. roqueforti* has been observed regarding properties such as colony appearance, aroma production, secondary metabolite production, and enzyme activity^2,7,12^. Of particular importance for cheese production is that strains can have different colony colours and textures, with commercial strains being sold partly on the basis of colour development. Studies with other fungal species have shown that colouration is due to the presence of pigments, which are an integral part of the coat wall of spores formed during colony growth. Fungal spore pigments generally belong to a class of secondary metabolites termed melanins, which are defined by their insolubility in water and organic solvents, and are generally negatively charged hydrophobic pigments with a high molecular weight that typically confer a dark hue^13^. Their detailed structure remains unknown due to difficulties in biochemical extraction and characterisation^14^. They are assembled predominately through either the dihydroxynaphthalene (DHN)-melanin (in ascomycete fungi such as *Aspergillus* and *Penicillium* spp.) or dihydroxyphenylalanine (DOPA)-melanin (in some basidiomycete fungi) pathways^13,15^, although other pathways are known such as Asp-melanin formation in *Aspergillus terreus*^16^, pyomelanin synthesis in *A. fumigatus*^17^, and catechol melanin production in *Agaricus bisporus*^18^. Melanin biosynthesis has been particularly well studied in the opportunistic fungal pathogen *Aspergillus fumigatus* where the main form of conidia-associated melanin is polyketide derived. Here, a polyketide synthase (PKS) catalyses the conversion of acetyl-CoA and malonyl-CoA into a naphtopyrone heptaketide intermediate, which is then truncated to the pentaketide 1,3,6,8-tetrahydroxynaphthalene (1,3,6,8-THN). This is followed by a series of reactions catalysed by reductases and dehydratases until 1,8-dihydroxynaphthalene (1,8-DHN) is formed, which is finally polymerised by a laccase leading to formation of the DHN-melanin pigment^19–21^. There is evidence of genomic clustering of melanin biosynthesis genes in various ascomycete genomes^22^. For example, in *A. fumigatus*, six linked genes involved in conidial pigment biosynthesis are clustered within a 19 kb region of the genome. These genes have been designated *alb1* (albino), *ayg1* (*Aspergillus* yellow-green), *arp1* (*Aspergillus* reddish-pink 1), *arp2* (*Aspergillus* reddish-pink 2), *abr1* (*Aspergillus* brown 1), and *abr2* (*Aspergillus* brown 2)^19^, based on changes of colony appearance in deletion mutants. In the opportunistic pathogen *Penicillium* (*Talaromyces*) *marneffei* a 25.3 kb melanin biosynthesis gene cluster has been detected with the same six genes contributing to DHN-melanin biosynthesis as in *A*. *fumigatus*^23^. Of significance to *P. roqueforti*, recent genome analyses of related species such as *Penicillium digitatum*, *Penicillium ucsense*, and *Penicillium oxalicum* also identified these six genes closely clustered in the genome, although not always in the same order or orientation^24,25^. This implies that the DHN-melanin biosynthesis pathway might be conserved across *Penicillium* species.

As well as pigment formation, both environmental and cheese production strains of *P. roqueforti* have been shown to have the potential to produce other secondary metabolites including mycotoxins such as roquefortine C, mycophenolic acid and PR toxin, particularly under favourable growth conditions^6,12,26^. However, they occur at sufficiently low enough concentrations in blue cheeses that they have been deemed a negligible risk to human health^27,28^. However, it has recently been reported that the melanin biosynthetic pathway itself can lead to production of certain mycotoxins^29^.

Given the iconic status of *P. roqueforti* as the ‘blue-cheese fungus’, the present study was undertaken to determine the genetic basis of pigment formation in the species with the aim to investigate whether it was possible to produce strains with new altered spore coat colours, which could be of great potential public and commercial appeal for cheese production. This was achieved by a combination of bioinformatic analyses, heterologous gene expression and targeted gene deletion studies to analyse the colour spectrum of spores that could be achieved. The effect of deletion of the pigment biosynthesis genes on mycotoxin and flavour volatile production was then determined. We finally used UV mutagenesis to produce non-genetically modified colour mutants and used selected strains for ‘non-blue’ cheese production as proof of principle.

## Results

### Identification of a putative DHN-melanin biosynthesis pathway in *Penicillium roqueforti*

**Fig. 1:**
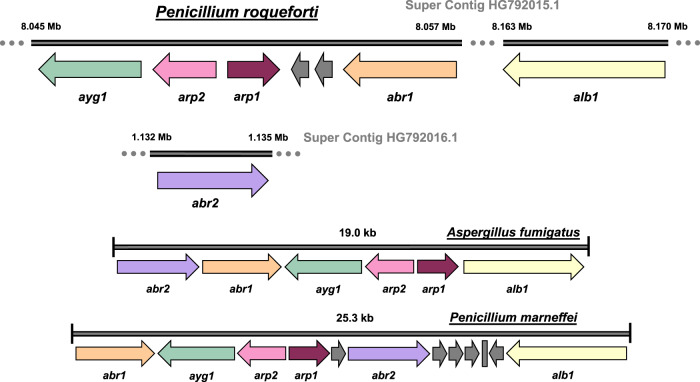
Clustering of the DHN-melanin biosynthesis gene assemblies in *Penicillium roqueforti* and closely related fungi (figure drawn to approximate scale). *Penicillium roqueforti* genes *ayg1* (Genbank CDM29601.1), *arp2* (CDM29602.1), *arp1* (CDM29603.1), *abr1* (CDM29606.1), and *alb1* (CDM29654.1) are all located on the same super contig HG792015.1. The laccase gene *abr2* (CDM30362.1) is located on a separate super contig HG792016.1 (top). By contrast, DHN-melanin gene clusters in *Aspergillus fumigatus* and *Penicillium marneffei* are tightly linked in their genomes (bottom). Other non-pathway genes are shown in grey.

Bioinformatic analyses were performed to determine whether the pathway genes required for the production of conidia-associated DHN-melanin were present in *P. roqueforti*. Homologues of all six target pathway proteins from *A. fumigatus* (Alb1, Ayg1, Arp1, Arp2, Abr1, and Abr2) were indeed identified in the *P. roqueforti* FM164 genome by BLASTP searches with *E* values < 0.01 for all proteins (Supplementary Fig. 1), and the same gene names were adopted for *P. roqueforti* (Fig. 1). The DNA sequences for the pathway genes in FM164 and the industrial cheese production isolate 74–88 were nearly identical, with no variation in the encoded amino acid sequences (GenBank accessions OQ680622-OQ680627). Thus, all genes for a functional DHN-melanin pathway were present in both the FM164 and 74–88 genomes of *P. roqueforti*. Five of the six genes (*alb1, ayg1, arp2, arp1*, and *abr1*) were found clustered on the same FM164 super contig HG792015.1 (Fig. 1). The genes *ayg1, arp2*, and *arp1*, were clustered and oriented in the same way across *P. roqueforti*, *A. fumigatus*, and *P. marneffei*. However, unlike *A. fumigatus* and *P. marneffei* the laccase gene *abr2* was located outside the five-gene cluster on a separate super contig HG792016.1. There was also a gap of over 100 kb between *alb1* and other members of the putative *P. roqueforti* melanin biosynthesis gene cluster (Fig. 1). Thus, despite some differences in the genomic organisation of individual genes, *P. roqueforti* revealed the genetic potential to produce a DHN-type melanin pigment.

### Enzyme inhibition

To further test whether a DHN-melanin pathway contributes to conidia pigmentation in *P. roqueforti*, known pathway enzyme inhibitors were applied to growing cultures. Even at the lowest concentration of 4 μg/mL, the addition of tricyclazole and pyroquilon resulted in reddish-pink-brown colonies (Supplementary Fig. 2). This was consistent with inhibition of hydroxynaphthalene reductases such as Arp2 and the presence of a functional DHN-melanin biosynthesis pathway in *P. roqueforti*.

### Heterologous expression of *alb1* and *ayg1* in *Aspergillus niger*

**Fig. 2:**
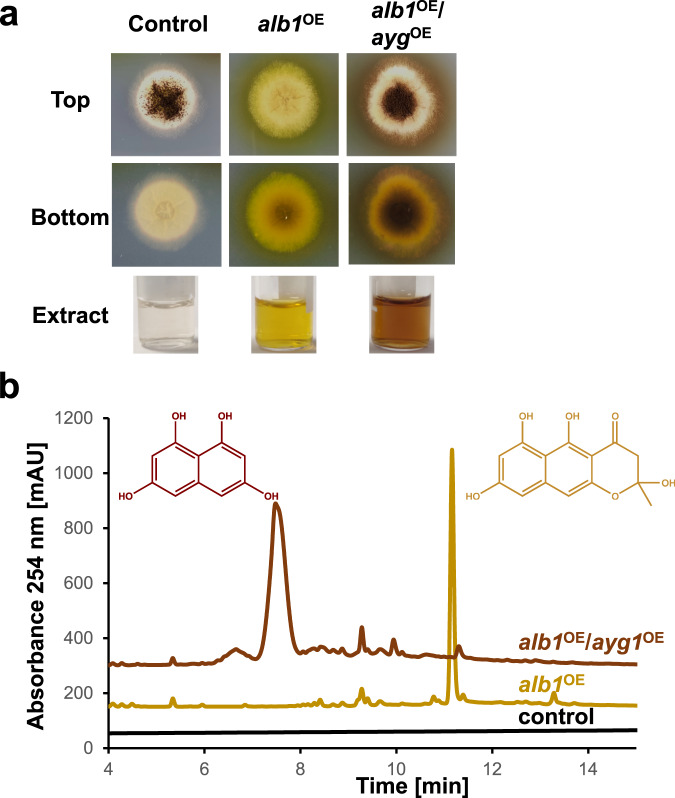
Heterologous gene expression and metabolite production in *Aspergillus niger*. **a** Colony phenotype and extracts from culture filtrates. The *A. niger* parental strain P2 (control) as well as the strains overexpressing the *P. roqueforti alb1* gene, either alone (*alb1*^OE^) or in combination with *ayg1* (*alb1*^OE^/*ayg1*^OE^), were incubated for 60 h on *Aspergillus* minimal medium and pictures were taken from top and bottom view. In parallel, strains were incubated for 40 h in liquid *Aspergillus* minimal medium and culture filtrate extracts are shown (Extract). **b** HPLC analysis of culture filtrate extracts shown in **a**. Exact-mass determination confirmed that the *alb1*^OE^ strain produces the naphthopyrone YWA1, which is converted into 1,3,6,8-tetrahydroxynaphthalene in the *alb1*^OE^/*ayg1*^OE^ strain.

To confirm that the putative DHN-melanin pathway in *P. roqueforti* followed that described for *A. fumigatus*, we constructed heterologous expression strains comprising the key polyketide synthase *alb1* either alone or in combination with *ayg1* in the *A. niger* expression platform strain P2 under regulatory control of the TerR-inducible *terA* promoter^30^. The growth of selected transformants was then compared to the parental strain on solid medium. The *alb1* overexpressing strain showed delayed conidiation but produced large quantities of a yellow metabolite that diffused into the medium, which was not observed in the parental strain (Fig. 2a). Furthermore, co-integration of *ayg1* into the *alb1* overexpressing strain resulted in more typical conidiation and led to a change from the yellow to a dark brown colouration that initially accumulated at the colony centre before diffusing into the medium (Fig. 2a). Cultivation of strains in liquid minimal medium resulted in a similar colour profile in culture extracts (Fig. 2a), noting that growth in submerged culture prevents conidia formation and therefore pigments were not attributable to the intrinsic melanin biosynthesis pathway of *A. niger*. UHPLC analysis of these extracts (Fig. 2b) showed the expected baseline profile for the parental strain, whereas the *alb1* overexpressing strain revealed a single major metabolite with an exact-mass *m/z* of 275.0504 [M – H]^−^ and sum formula of C_14_H_12_O_6_, in perfect agreement with production of the heptaketide YWA1 (theoretical *m/z* = 276.0634). When *alb1* was co-expressed with *P. roqueforti ayg1*, a new metabolite was produced (Fig. 2b) with an exact-mass *m/z* of 191.0372 [M – H]^−^ and sum formula C_10_H_8_O_4_, in perfect agreement with the formation of the pentaketide 1,3,6,8-tetrahydroxynaphtalene (theoretical mass m/z of 192.0423) from YWA1. These results confirmed that *alb1* and *ayg1* produce functional enzymes involved with synthesis of intermediates in the DHN-melanin biosynthesis pathway and were, therefore, likely to be involved in the overall formation of DHN-melanin.

### Deletion of DHN-melanin pathway pigment biosynthetic genes in *Penicillium roqueforti*

**Fig. 3:**
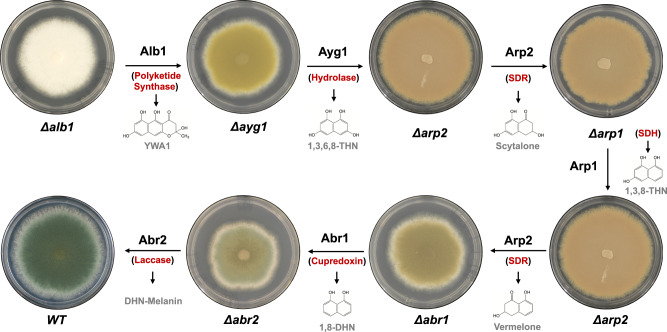
Proposed DHN-melanin biosynthesis pathway in *Pencillium roqueforti* showing genes involved and their respective putative products. Figure also shows the impact of gene deletion on colony pigmentation in representative transformant strains. *Δalb1* (albino), *Δayg1* (yellowish-green), *Δarp1* (reddish-pink), *Δarp2* (reddish-pink), *Δabr1* (brown), and *Δabr2* (brown) depicted at each step in the DHN-melanin production process. The predicted gene products are in red, and putative resulting compounds are shown in grey. SDR= short-chain dehydrogenase/reductase, SDH scytalone dehydratase.

To confirm the contribution of all six genes of the putative DHN-melanin biosynthesis pathway in *P. roqueforti*, we developed a transformation protocol for targeted gene deletion involving gene replacement by the hygromycin B resistance cassette. The success rate of homologous recombination into target loci varied between 4.8–12.7%, with between 1–8 deletion mutants obtained for each respective gene (Supplementary Table 1; Supplementary Fig. 3). The resulting transformants maintained conidia production whilst displaying alterations in pigmentation (Fig. 3). The *Δalb1* deletion mutants produced an all-white colony phenotype, in agreement with the lack of YWA1 production as an essential precursor in DHN-melanin biosynthesis. The *Δayg1* deletants produced yellowish-green, *Δarp1* reddish-pink-brown, *Δarp2* reddish-pink-brown, *Δabr1* brown, and *Δabr2* brown colonies (Fig. 3). Positional PCR confirmed the correct insertion for gene replacement in representative transformants (Supplementary Fig. 4). To further ensure accuracy of gene deletions, genes adjacent to the targeted site were sequenced which confirmed that no further mutations had occurred^31^. In follow-up complementation studies, both the c*Δalb1* and c*Δabr2* genes were successfully re-integrated into the respective *Δalb1* and *Δabr2* loci. This resulted in a rescue phenotype with return to wild-type pigment production (Supplementary Fig. 5). Positional PCR again showed correct integrations (Supplementary Fig. 6).

Taken together, results confirmed that all six targeted genes were involved in pigment formation and that pigment biosynthesis seemed to follow the same pathway identified from *A. fumigatus*^19^. Importantly, the striking change in colony appearance of deletion mutants implied that it might be feasible via pathway disruption to generate strains of *P. roqueforti* displaying different spore coat colours that might be used in cheese manufacturing.

### Impact of melanin pathway gene deletion on roquefortine C and mycophenolic acid production

**Fig. 4:**
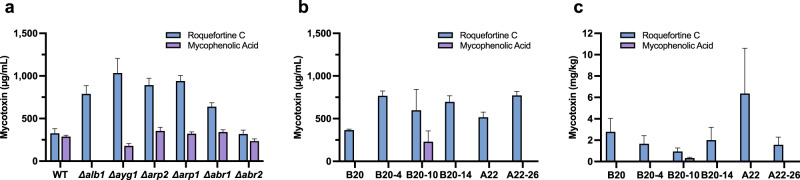
Production of the mycotoxins roquefortine C and mycophenolic acid by various strains of *Penicillium roqueforti*. **a** Sucrose-induced mycotoxin levels of the parental isolate 74–88 (WT) compared to various DHN-melanin biosynthesis gene deletion strains (*Δalb1, Δayg1, Δarp1, Δarp2, Δabr1* and *Δabr2*). **b** Sucose-induced mycotoxin levels of ascospore isolates B20 and A22 as compared to UV-induced colour mutant strains B20-4, B20-10, B20-14 and A22–26. B20 and derived isolates show a significant variation in roquefortine C production (one-way ANOVA; *F* = 5.4, df = 3,8, *P* = 0.03). A22 and mutant A22–26 also show a significant variation in roquefortine C production (two tailed *T* test; *T* = 5.8, df = 4, *P* = 0.004). **c** Mycotoxin levels in trial cheeses of ascospore isolates B20 and A22 as compared to UV-induced colour mutant strains B20-4, B20-10, B20-14 and A22–26. Note that assay conditions for **a** and **b** were mycotoxins recovered from six agar plugs and resuspended in 400 µl methanol before final application of 5 µl aliquots to HPLC column, whereas data from **c** is expressed as recovery from cheese wheels. Error bars indicate ±SD.

A prerequisite for using colour mutants for ‘non-blue’ mould-ripened cheese production is that such strains do not display excessive activation of secondary metabolite toxin production. Therefore, we compared mycotoxin production in the pigment deletion strains with the parental strain 74–88 under in vitro conditions using sucrose as the main carbon source, which favours mycotoxin production. HPLC analyses showed that 74–88 produced both mycophenolic acid (MPA; 289 ± 16 µg/mL) and roquefortine C (326 ± 54 µg/mL) under the test conditions (Fig. 4a). There was no overall clear change in MPA production between 74–88 and the gene deletion strains except for the *Δalb1* mutant, in which there was negligible MPA production (one way ANOVA: *F* = 71.8; *DF* = 6,14; *p* < 0.0001). By contrast, there was a significant overall increase in roquefortine C production in most gene deletion strains (one way ANOVA: *F* = 31.3; *DF* = 6,14; *p* < 0.0001) (Fig. 4a). The highest levels were seen in the *Δalb1*, *Δayg1*, *Δarp1*, *Δarp2,* and *Δabr1* strains, which showed between 1.9- to 3.1-fold increases compared to the 74–88 parent. The *Δarb2* strain showed little difference to the control. Despite the production of mycotoxins under these conditions it should be noted that mycotoxin production during blue-cheese ripening is at a much lower or negligible rate^27,28^, and therefore, such levels of increase in roquefortine C were not expected to impact on cheese production.

### Detection of flavour volatiles in pigment biosynthesis mutants

**Fig. 5:**
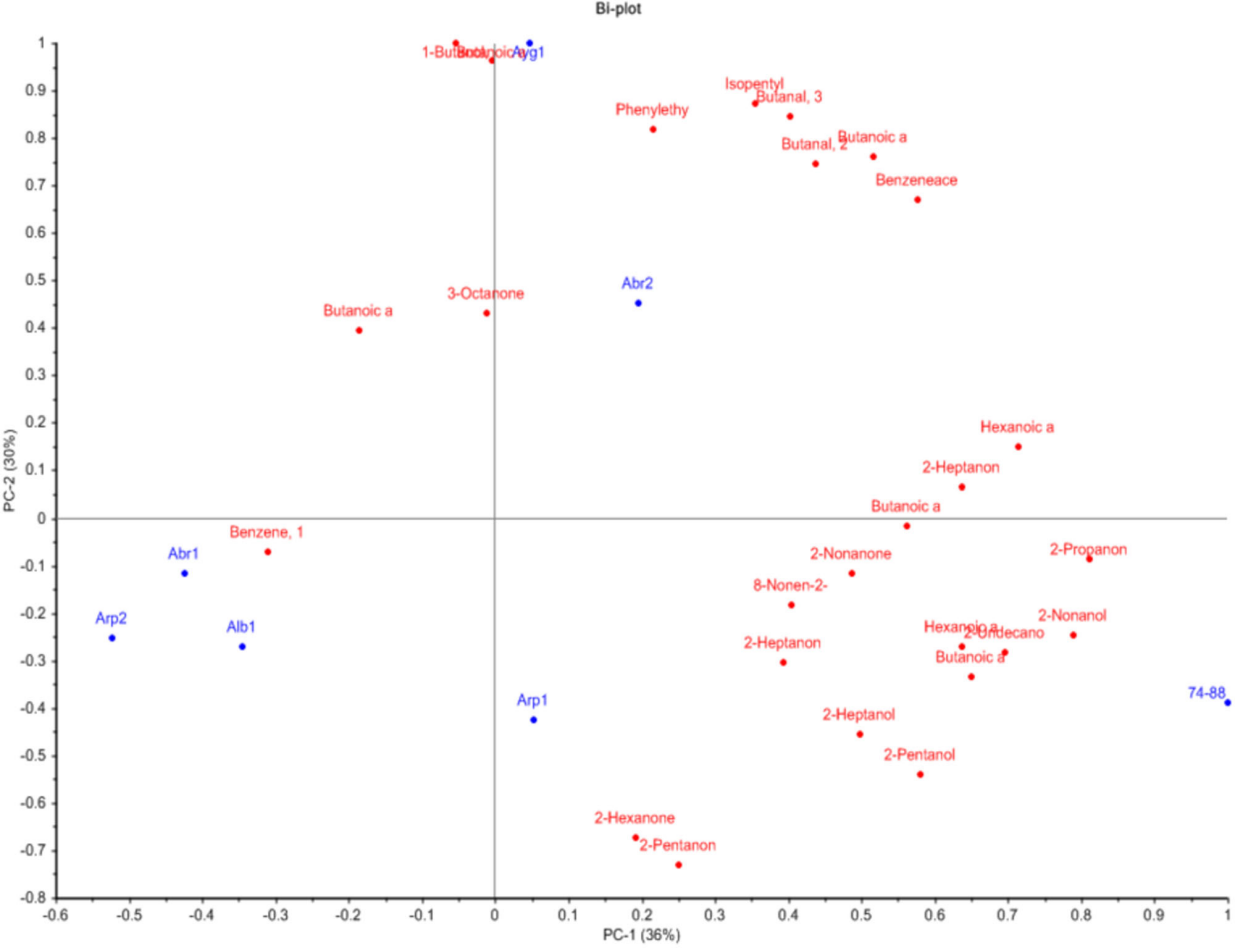
PCA plot. Differences in profiles of production of volatile compounds (displayed in red) between the parental strain 74–88 and various DHN-melanin biosynthesis gene deletion strains (*Δalb1, Δayg1, Δarp1, Δarp2, Δabr1* and *Δabr2*) (displayed in blue).

The production of aroma volatiles by *P. roqueforti* is essential as it contributes to the typical taste of blue cheeses. Therefore, we performed SPME-GCMS analysis on the pigment biosynthesis mutants focusing on 26 compounds previously associated with blue-cheese aroma^32,33^. Principal component analysis (PCA) revealed differences in volatile profiles between strains (Fig. 5). The various gene deletion mutants all differed from the parental 74–88, although some (e.g., *Δalb1*, *Δapr2* and *Δabr1*) clustered in close proximity to each other. Importantly, gene deletion had little effect on the production of ketones that are mainly associated with blue-cheese flavour (e.g., 2-pentanone, 2-hexanone, 2-heptanone, 3-octanone, 8-nonen-2-one, 2-nonanone). Levels of butanal-3-methyl, butanal-2-methyl, butanoic acid-3-methyl, 2-hexanone, 2-heptanone, 8-nonen-2-one, benzene-1-methoxy-4-methyl and benzeneacetaldehyde were also not clearly affected. By contrast, certain esters (butanoic acid ethyl ester, hexanoic acid ethyl ester) and alcohols (2-pentanol, 2-heptanol, 2-nonanol) were present at lower levels in all deletion strains (Supplementary Table 3), which could have some impact on the taste of cheese produced with such strains.

### UV colour mutants and ‘colour-cheese’ production

**Fig. 6:**
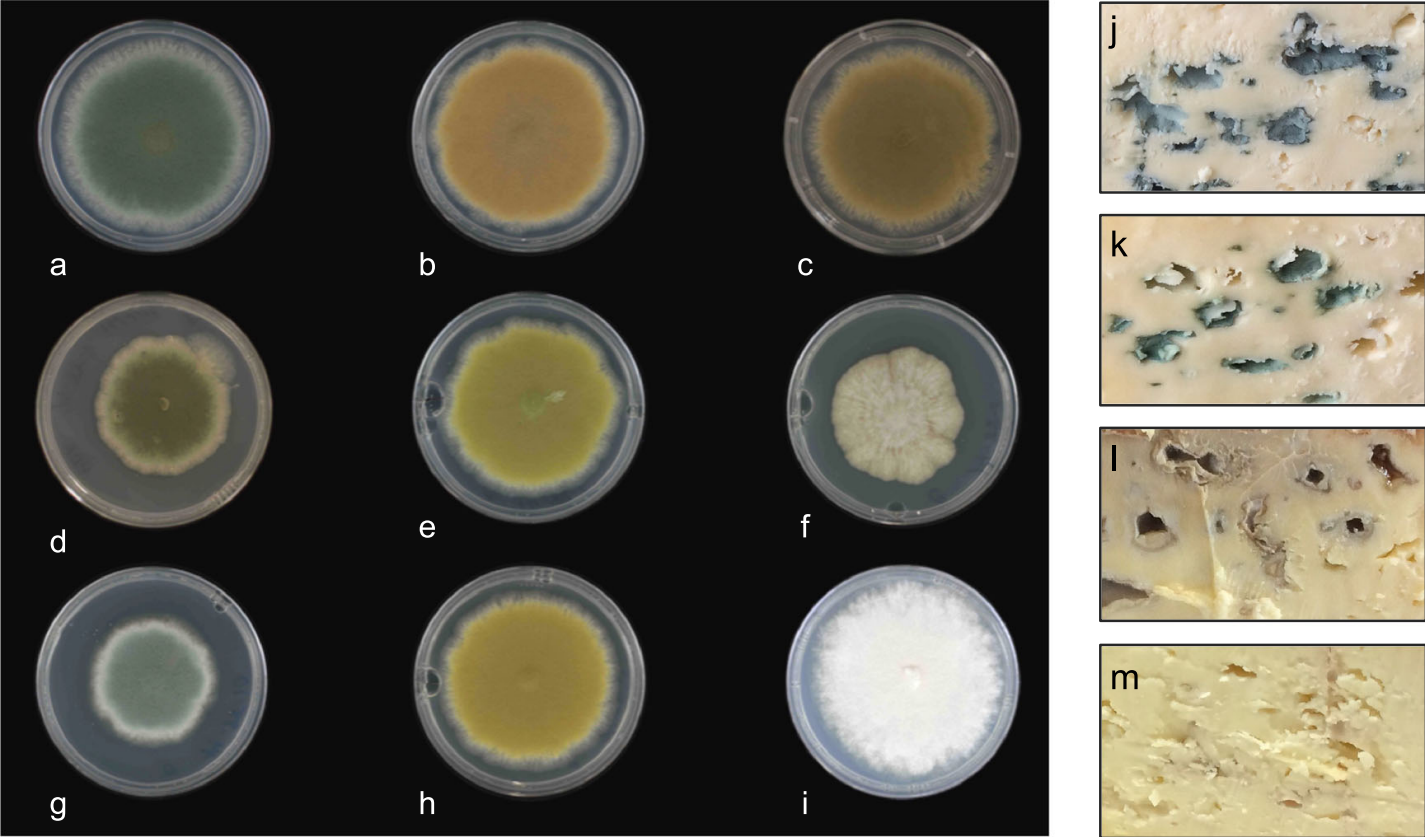
UV colour mutant strains and their applications in cheese production. Wild-type and representative UV colour mutant strains of *Pencillium roqueforti*: **a** Parental (wild type) 74–88, **b** 74-88-1 (*arp2*-T290A), **c** 74-88-2 (*abr1*-Y42*;D43N), **d** 74-88-4 **e** 74-88-5 (*ayg1*-L258P), **f** 74-88-6, **g** 74-88-10, **h** 74-88-12 (*ayg1*-C344R), **i** B20-1 (*alb1*-L102S). Cross sections of cheeses made with parental and UV colour derivatives (mutants) of *Penicillium roqueforti* strain B20: **j** Parental wild-type B20, **k** strain B20-14 (green), **l** strain B20-10 (fawn), **m** strain B20-4 (albino/white). All cheeses matured for 12 weeks to allow sporulation and colour development.

It was not possible to use the gene deletion strains for cheese production in a commercial setting given current food regulatory legislation. Therefore to exploit the colour change in DHN-melanin biosynthesis mutants, classical UV mutagenesis was instead used to successfully produce multiple colour mutants of *P. roqueforti* isolate 74–88 and the ascospore-derived B20 and A22 strains (Fig. 6; Supplementary Fig. 7). DNA sequences of all six DHN-melanin pathway genes of selected 74–88-derived UV colour mutants were compared to those present in the wild-type parent. The same pathway genes were also sequenced from selected B20 and A22 colour mutants and compared to the B20 and A22 parental sequences. The colour mutants 74–88–1 (brown), 74–88–2 (olive brown), 74-88-5 (green), 74-88-12 (mustard fawn) and B20-1 (white/albino) (colony colours in parentheses) were found to possess specific mutations in the genes corresponding to their phenotypic colouration within the DHN-melanin pathway, namely 74-88-1 (*arp2*-T290A), 74-88-2 (*abr1*-Y42*;D43N), 74-88-5 (*ayg1*-L258P), 74-88-12 (*ayg1*-C344R), and B20-1 (*alb1*-L102S) (Supplementary Table 2, Fig. 6). Comparison of colony phenotypes between the gene deletion and the UV mutants with the same effected gene revealed broadly the same colouration. However, there were subtle differences in hue between some of the strains, particularly for the *arp*2 gene mutants (Supplementary Fig. 8). Growth of 74-88-1 (*arp2*-T290A) on media supplemented with either of the DHN-melanin pathway enzyme inhibitors tricyclazole or pyroquilon resulted in a negligible change in colouration, consistent with a lack of hydroxynaphthalene reductase activity (Supplementary Fig. 9). All other colour mutant strains [74-88-4 (greyish green), 74-88-6 (white), 74-88-10 (intense blue), A22-26 (powder blue)] showed no mutation in the coding region of any of the six genes and were genetically identical in the DHN-melanin pathway to the parent (Supplementary Table 2). This implied other UV mutational changes were affecting pigment production in these mutants.

Importantly, GCMS analysis was again used to test any possible impact on flavour volatile production in the UV colour mutant strains. Analysis of the B20 parent compared to eight UV colour mutants revealed that most mutants clustered close to the B20 parent, although one strain (B20-16) was more divergent (Supplementary Fig. 10). By contrast, analysis of the A22 parent compared to 19 UV colour mutants revealed that most mutants clustered into two groups (one with the A22 parent), although one strain (A22–36) was more divergent (Supplementary Fig. 11).

Certain UV colour mutant strains [B20–4 (white/albino); B20-10 (fawn); B20-14 (green); A22–26 (powder blue)] were then tested for sucrose-induced production of mycotoxins compared to the parental strains (B20 and A22). Between a 1.4 to 2.0-fold increase in roquefortine C production in the colour mutants was observed (Fig. 4b), but with levels still comparable or below the 74–88 production and gene deletant strains (Fig. 4a). Neither the B20 nor A22 parent produced detectable MPA under the test conditions, although MPA was found at low levels in one colour mutant B20–10. The same strains (B20–4, B20–10, B20–14, A22–26) were then chosen for the production of “non-blue” cheese to test suitability in cheese manufacture. The use of the colour mutants markedly changed the appearance of the resulting cheese compared to the parental strains, with different colours evident in the cavities of the cheese, which were consistent with the spore colours produced in vitro by each strain (Fig. 6). Subsequent UHPLC analysis revealed comparable or lower levels of roquefortine C in cheeses made with the UV colour mutants compared to cheeses made with the parental isolates (Fig. 4c), although the colour mutant B20–10 again showed low-level MPA production. These results clearly indicated that the DHN-melanin biosynthesis pathway is a suitable target to change the appearance of traditional ‘blue-cheese’ fungal strains without significantly increasing mycotoxin levels in the cheese product.

## Discussion

*Penicillium roqueforti* has a central role in the production of interior mould-ripened cheeses such as Gorgonzola, Roquefort, and Stilton where the fungus is critical for flavour and texture development through its enzymatic activity. In addition, asexual sporulation of the fungus in cavities of the cheese results in the characteristic blue-veined appearance, with commercial strains being sold partly on the basis of colour development. Variation in both spore colour and colony texture has been described for worldwide isolates from cheese and non-cheese substrates^2,7^. Despite the importance of colour in *P. roqueforti*, the genetic basis of spore pigmentation in this species has not previously been elucidated. In the present study we drew on knowledge of pigment development in other ascomycete fungi^19–21^ to identify a DHN-melanin biosynthetic pathway in *P. roqueforti* and used experimental approaches to confirm the functional activity of the pathway. Results also demonstrated the possibility to generate new coloured strains via pathway disruption, of potential public and commercial appeal, which was further confirmed by the use of UV mutagenesis to induce a range of colour mutants.

The blue-green appearance of *P. roqueforti* conidia suggested that a DHN-melanin biosynthesis pathway like that described for *A. fumigatus*^19^ might be present. The availability of the *P. roqueforti* FM164 genome^8^ allowed BLAST analyses, which led to identification of a canonical DHN-melanin biosynthetic pathway in *P. roqueforti*, comprised of six genes (*alb1, ayg1, arp2, arp1*, *abr1* and *abr2*) whose sequential enzyme activity has previously been shown to lead to synthesis of DHN-melanin^13,19,22–24^. The same set of genes were detected and sequenced from the 74–88 industrial isolate of *P. roqueforti* used for experimental work in the present study. These results are consistent with reports of the presence of DHN-melanin pathways in related *Penicillium* and *Aspergillus* species, which show evidence of genome clustering of pathway genes thought to be linked to pathway evolution and regulation^20–22,24,25^. However, *P. roqueforti* FM164 is unique amongst so far reported penicilia in that the laccase gene *abr2* is located on a separate super contig (HG792016.1) from the other five clustered pathway genes, whilst there was a relatively large distance (over 100 kb) between *alb1* and the four other members of the main cluster. Such separation of DHN-melanin pathway genes has also been observed in *Botrytis cinerea* and *Alternaria alternata* although any functional significance is unclear^29^.

A combination of biochemical enzyme inhibition, heterologous gene expression, and gene modification (GM) approaches were applied to evaluate whether the putative DHN-melanin biosynthetic pathway was indeed functional in *P. roqueforti*. First, known enzyme inhibitors of the DHN-melanin pathway were used to confirm gene function^34,35^. Addition of the Arp2 inhibitors tricyclazole and pyroquilon resulted in the production of reddish-pink-brown colonies, consistent with *arp2* being a key component in the DHN-melanin pathway of *P. roqueforti*.

Second, the first two putative pathways genes *P. roqueforti alb1* and *ayg1* were expressed in an *A. niger* heterologous expression system^16,30^ to identify the metabolites produced. Transformants expressing *alb1* grown on solid media showed delayed conidation of the host *A. niger* strain, most likely due to high-level production and potentially intracellular accumulation of the naphthopyrone YWA1 consistent with the yellowish appearance of the mycelium. This effect was relieved in transformants expressing both *alb1* and *ayg1* on solid media, thought to be due to better secretion of the tertrahydroxynaphtalene compared to YWA1 as the mycelium of the latter strains appeared white. When grown in liquid culture, a strain expressing the *P. roqueforti alb1* gene was found to produce the heptaketide YWA1, and when *alb1* was co-expressed with *P. roqueforti ayg1* then 1,3,6,8-THN was produced. These results not only confirmed that the genes encode functional proteins, but also demonstrated that these key first stages of pigment biosynthesis in *P. roqueforti* follow the DHN-melanin biosynthesis pathway described for *A. fumigatus*^19^. It is noted that pigment formation could be attributed solely to expression of the *P. roqueforti alb1* and *ayg1* genes because the endogenous DHN-melanin pathway of *A. niger*, including formation of the black pigment aspergillin^20,36^, is not expressed under the liquid growth conditions of the assay. This result is consistent with previous studies on heterologous expression of fungal melanin pigments in *Aspergillus oryzae*, which also used a wild-type strain in terms of conidial pigment production, and no interference from *A. oryzae* conidial pigments was observed^37^. It is also noted that heterologous expression of further elements of the pathway was considered problematic due to the requirement for multiple gene constructs and need for suitable cellular localisation of later stages of the pathway, and was therefore not pursued.

Third, deletion cassettes were successfully integrated into the genome to individually remove all six putative DHN-melanin pathway genes. Gene deletion resulted in a dramatic change in conidial pigmentation for each step in the pathway. Removal of the *alb1* gene produced white (albino) colonies with conidia that lacked any visible pigmentation, indicating lack of polyketide synthesis as described in other *Aspergillus* and *Penicillium* species^19,23^. Deletion of *ayg1* resulted in yellowish-green colonies, most likely due to the accumulation of the heptakide YWA1 and other *alb1* intermediate products. Deletion of *arp2* and *arp1* each resulted in reddish-pink-brown colonies, likely due to the accumulation of 1,3,6,8-THN and scytalone, respectively. Deletion of *abr1* and the final laccase *abr2* gene both resulted in colonies with a brown colouration, likely linked to accumulation of vermelone or 1,8-DHN and other pathway intermediates, respectively^15,19,23^. Whilst results were consistent with previous reports and the heterologous expression studies, confirmation of the putative chemical intermediates will require further biochemical work. Indeed, deletion of genes in the DHN-melanin biosynthetic pathway can lead to several alternative pathway byproducts such as formed by auto-oxidation rather than enzyme action alone^35^. To further confirm gene function, complementation studies were undertaken with the polyketide synthase (*alb1*) and laccase (*abr2*) genes as representatives at the start and end of the DHN-melanin pathway. In both cases, rescue with the parental gene restored wild-type conidial pigmentation in the complemented strains.

It was realised that the ability to produce colour derivative strains of *P. roqueforti* via disruption of the pigment biosynthetic pathway provided the exciting possibility of producing new commercial strains of the fungus with alternative spore colours rather than just the traditional blue-green colouration. However, it was important to understand any possible impact of disruption of the DHN-melanin pathway on the physiology of such colour mutants. Therefore, studies were undertaken to assess the impact on mycotoxin levels and flavour volatile production in the various gene deletion strains.

Regarding mycotoxins, the impact on mycophenolic acid (MPA) and roquefortine C was investigated as two key secondary metabolites of safety concern^27,28^. There was no significant increase in MPA production between the 74–88 parent and the gene deletant strains. Perhaps surprisingly, a significant increase in roquefortine C production was observed in many of the gene deletion strains, in particular a ca. threefold increase in the *Δayg1* strain. The reasons for this increase were unclear given that regulation of secondary metabolite pathways are complex. It has recently been shown in *Alternaria alternata* that the biosynthetic pathway for altertoxin production utilises many of the enzymes of the DHN-melanin pathway, indicating this pathway as a source for fungal toxin production^29^. Despite the increased roquefortine C levels observed in certain DHN-melanin gene deletants, it is important to note that levels still remained relatively low and of the same overall magnitude as the 74–88 production strain. Furthermore, production was induced by the presence of sucrose, which is absent during cheese production. Other abiotic factors such as temperature, NaCl concentration, and pH can also influence mycotoxin production in *P. roqueforti*^38^.

Regarding volatile production, the impact of gene deletion was investigated on 26 different flavour compounds associated with ripening and aroma development in blue cheese^32,33^. No significant change in the levels of ketones, principally associated with blue-cheese flavour, was seen in any of the deletion strains. By contrast, lower levels of certain esters and 2-alcohols were found in all the deletion strains. The reason(s) for these lower levels was unclear, although it is speculated this may be due to build up of pathway intermediates. Results, therefore suggest that DHN-melanin pathway mutants of *P. roqueforti* might have slightly altered aroma profiles if used in cheese production whilst retaining the key ‘blue-cheese’ flavour. However, it is cautioned that the present study used a model milk system for volatile production^32,33^ and results cannot be directly extrapolated to whole cheese production. Elsewhere there has been reported to be much variation in flavour volatile production between isolates of *P. roqueforti*^12^.

Encouraged by these results, we then investigated if it were possible to create pigment mutants of *P. roqueforti* by classical UV mutagenesis given that it is not possible to utilise GM strains in commercial cheese manufacture under current food legislation, but UV-derivatives are allowed. Indeed, a UV-derived white-spored strain of *P. roqueforti* has previously been commercialised^39,40^. A series of colour mutants were successfully produced following UV irradiation of the 74–88 production or B20 and A22 ascospore-derived strains. For most colour mutants, those producing colonies with a particular non-standard colour phenotype were found to contain UV-induced mutations in the corresponding gene in the DHN-melanin pathway. For example, the reddish-brown colour mutant 74-88-1 had a mutation in the *arp2* gene (T290A), the green mutant 74-88-5 had a mutation in the *ayg1* gene (L258P), and the white (albino) mutant B20-1 had a mutation in the *alb1* gene (L102S). However, interestingly the hue and depth of colour of the UV mutants sometimes differed slightly from the respective whole gene deletant strain. This suggested that some of the UV-mutated genes might still be producing a protein product with partial functionality, although the impact of UV mutations elsewhere in the genome cannot be discounted. Furthermore, some colour mutants (e.g., greyish green and intense blue) were produced for which no mutation could be found in any of the coding regions of the DHN-melanin pathway genes. This indicates that elements in the promoter region and/or genetic elements other than the DHN-melanin pathway can mediate pigment production and colour in *P. roqueforti*. Thus, UV mutagenesis might enable the generation of a wider spectrum of colour strains than targeted DHN-melanin pathway gene deletion alone. It is noted that slightly different pathways for DHN-melanin synthesis have been reported for *Aspergillus nidulans* and *Aspergillus flavus*^20^ and that a non-canonical melanin biosynthetic pathway has been described from *A. terreus*^16^, as well as synthesis of pyomelanin in *A. fumigatus*^17^.

Certain colour derivative mutants were then used as starter cultures in cheese production. The arising cheeses indeed showed striking differences in the colour of veining of the cheese compared to the parental strain. Levels of mycotoxins produced in cheeses by colour derivatives were comparable or lower than those produced by the parental isolates, indicating likely safe use for food production although this finding will require confirmation with a larger number of strains. This was also consistent with results of mycotoxin production on sucrose-inducing media by representative UV colour mutants where levels were comparable or below the 74–88 production strain, again indicating safe use for food production. It is noted that mycotoxins have previously been reported in blue cheeses, but the levels produced are not considered a health risk, and mycotoxins may be unstable in the cheese matrix^28,41^. However, it is cautioned that *P. roqueforti* can produce additional mycotoxins, which were not examined in the present study^6,12^.

Although *P. roqueforti* is best known for applications in cheese production, the species is a relatively common saprotrophic mould found on various natural substrates^6^. Therefore, the presence of the DHN-melanin pigment in the spore wall can be explained partly by the fact that melanin can provide protection against environmental UV radiation^13,35,42^. Indeed, we found that deletion of representative genes from the DHN-melanin biosynthetic pathway (*Δalb1, Δayg1* and *Δarp1*) resulted in significantly lower spore survival than those of the parental 74–88 isolate when exposed to UV stress (Supplementary Fig. 12), consistent with parallel findings on *P. roqueforti* melanin-deficient conidia exposed to UV radiation^42^. In addition, fungal melanins may confer protection against oxidative and temperature stress^35^ and are required as structural components for correct assembly of the fungal cell wall^14^.

To conclude, work presented in this study has revealed the principal pigment pathway used by *P. roqueforti* for production of DHN-melanin, involving a canonical set of six genes (*alb1, ayg1, arp2, arp1*, *abr1* and *abr2*) most of which were clustered together in the genome. Disruption of pathway genes by gene deletion or UV mutagenesis resulted in strains producing spores with altered pigmentation. Such strains might be of public and commercial interest for manufacture of new ‘non-blue’ coloured mould-ripened cheese and represent a further step in the domestication of the species. Market acceptance will reveal the future of such products. UV studies indicated that additional genetic elements other than the DHN-melanin pathway alone can also mediate pigment production and colour in *P. roqueforti*. Future genome sequencing of such non-standard colour mutants of *P. roqueforti* is envisaged to provide insights into additional pigment pathways.

The present study also found that deletion of certain genes of the DHN-melanin pathway of *P. roqueforti* resulted in unexpected changes in both the production of the mycotoxin roquefortine C and also certain flavour volatile compounds. This warrants further work to investigate links between DHN-melanin biosynthesis and other cellular processes.

## Methods

### Strains and media

The main *Penicillium roqueforti* isolate used was 74–88 [wild-type strain isolated from DANISCO PS stock^9^]. Two ascospore-derived strains B20 and A22 (trialled in cheese manufacture) were also used in UV mutagenesis work (all strains held in the BDUN University of Nottingham culture collection). Potato dextrose agar (PDA) [39 g Oxoid (UK) PDA per litre] was the principal media used for strain cultivation and characterisation of pigment production, following media screening by Cleere^31^. Liquid cultures were cultivated using *Aspergillus* complete media broth (ACMB)^43^. *Aspergillus niger* strain P2 and transformants were routinely cultivated at 28 °C on *Aspergillus* minimal medium^30^ with either 50 mM glucose and 10 mM glutamine (GG10) or 100 mM glucose and 20 mM glutamine (GG20) as carbon and nitrogen sources. Working stocks of fungal strains were cultured at 28 °C on PDA slopes and were kept in 10% glycerol under liquid nitrogen for long-term storage. Spore suspensions were prepared by growth on PDA slopes at 28 °C for 5–7 days before harvest using 0.1% sterile tween 20 (Sigma, UK), filtering through Miracloth (Calbiochem, UK) and counting using an improved Neubauer haemocytometer. *Escherichia coli* strains used for genetic transformations were either DH5α or XL1-Blue (Stratagene, USA).

Further strains of *P. roqueforti* were produced by UV mutagenesis under conditions estimated to result in 5–10% spore survival (Supplementary Fig. 13). UV-treated spore suspensions were stored in the dark at 4 °C for 1 hr prior to plating spores on 12 cm diameter plates containing PDA. Plates were inspected from 3 days onwards for the appearance of colonies with non-standard pigmentation. Colouration was scored using a colour guide^44^.

For cheese production, 5 ml of inoculum (1 × 10^9^ spores/mL) of relevant strains was supplied to Moydens Cheese Ltd. (UK) or Highland Fine Cheeses Ltd. (UK) who prepared cheese wheels according to proprietary recipes used for commercial blue-cheese production.

### Bioinformatic analysis

AF293 sequences of the Alb1, Ayg1, Arp1, Arp2, Abr1, and Abr2 proteins and corresponding genes involved in the conidial pigmentation of *A. fumigatus*^19^ were obtained via the AspGD website (http://www.aspgd.org/) and stored using MacVector 12 (MacVector, UK). Sequences were used in unidirectional BLASTP searches against the FM164 genome^8^ to obtain putative homologous genes from *P. roqueforti* (http://fungi.ensembl.org/Penicillium_roqueforti_fm164_gca_000513255/Info/Index). In order to verify whether the same DNH-melanin genes were present in *P. roqueforti* 74–88, PCR and gene sequencing were performed as described below. Sequences from the reference FM164 genome were used to design sets of oligonucleotide primers to amplify and sequence the same putative melanin biosynthetic genes from isolate 74–88 using MacVector 12 or manual design (Supplementary Table 4).

### Genomic DNA extraction, PCR, gel electrophoresis and DNA sequencing

Genomic DNA was extracted using a phenol-chloroform method^45^ with the modification that phase-lock tubes (VWR, UK) were used to improve separation of the phenol-chloroform and aqueous layers. DNA concentrations here and elsewhere were measured using an Implen P330 Geneflow NanoPhotometer (Implen, Germany).

PCR was performed using Phusion High Fidelity DNA Polymerase (New England Biolabs, UK) utilising a Techne (UK) thermo cycler. DNA samples were resolved in TAE agarose gels (0.7%–1.5% w/v) supplemented with 100 μL/L of 1 mg/mL ethidium bromide. Individual gels show DNA samples all derived from the same experiment that were processed in parallel. PCR products were purified using a Macherey-Nagel PCR cleanup kit (Fisher Scientific, UK). Amplicons were sequenced by the Queen’s Medical Centre sequencing lab (University of Nottingham, UK). Sequences were processed using MacVector 12 and aligned using Multalign (http://multalin.toulouse.inra.fr/multalin/).

### Enzyme inhibitors

The chemicals tricyclazole and pyroquilon, previously reported to inhibit hydroxynaphthalene reductases involved in the DHN-melanin biosynthetic pathway^34^, were obtained from Sigma (UK) and dissolved in ethanol at a stock concentration of 3 mg/mL. Tricyclazole and pyroquilon were then diluted to working concentrations of 30, 15, 8 and 4 μg/mL in PDA growth media. Isolate 74–88 was assayed on media supplemented at each concentration, while later trials with 74–88 derivatives used only supplementation at 30 μg/mL (all tests in triplicate with ~500 spores per plate). Plates were incubated at 28 °C for 7 days. Ethanol-supplemented controls were included.

### Heterologous expression of *alb1* and *ayg1* in *Aspergillus niger*

To identify the metabolites produced by *P. roqueforti* Alb1 either alone or in combination with Ayg1, the respective genes were amplified using Phusion High Fidelity DNA Polymerase and genomic DNA of 74–88 as a template. The 6.4 kb *alb1* gene was amplified with oligonucleotides tagX_Proq_PksP_fw and Proq_PksP_tag_rv, and the 1.45 kb *ayg1* gene with oligonucleotides IF_Ayg1_TagSMx_f and IF_Ayg1_TagSMx_rv (Supplementary Table 4). Oligonucleotides contained compatible overhangs to the 5’- and 3’-end of the *Nco*I cloning site of the his_SM-Xpress expression plasmid^16^, adding an N-terminal His-tag to the gene sequence and controlling gene expression from the TerR-inducible *terA* promoter in the *A. niger* expression platform strain P2^30^. In vitro recombination by the InFusion HD cloning kit (TaKaRa/Clontech, UK) was used to integrate genes into plasmids. A plasmid with the phleomycin resistance cassette (*ble*) was used for *alb1* and with the hygromycin resistance cassette (*hph*) for *ayg1*^16^. Plasmids were amplified in *E. coli* DH5α and isolation of the *ayg1*-containing plasmid was performed with a Machery-Nagel NucleoSpin plasmid isolation kit (Fisher Scientific, UK) and isolation of the *alb1*-containing plasmid with a Plasmid Midi kit (Qiagen, UK).

Transformation of the *A. niger* P2 strain was performed according to Geib and Brock^46^. First, the *A. niger* P2 strain was transformed with the *alb1*-containing expression plasmid using 80 µg/mL of phleomycin as a selection marker, with subsequent cultivation of selected transformants on GG10 medium containing 40 µg/mL phleomycin. One of the *alb1* overexpressing strains that showed visible production of a new pigment absent from the parental P2 strain was then selected for subsequent transformation with the *ayg1-*containing plasmid using 140 µg/mL hygromycin B as a selection marker. Transformants were analysed for metabolite production by inoculation of 25 mL of liquid GG20 medium with 1 × 10^6^ conidia/mL followed by incubation for 40 h on a rotary shaker (150 rpm) at 28 °C. Extracts from culture supernatants and mycelia were prepared as previously described^47^ and subjected to HPLC analysis on a Dionex UltiMate 3000 (Thermo-Fisher Scientific, UK) system using an analytical Eclipse XDB C18 (Agilent, 250 mm × 4.6 mm, particle size 5 µm) column^16^. High-resolution exact-mass determination was performed in negative mode on a Bruker impact II Ultra-High-Resolution LC–QTOF MS with an Eclipse XDB C18 column as specified above^47^. To visualise production of coloured metabolites from transformants, a minimal medium plate was point inoculated with selected strains and incubated for 60 h at 28 °C before photographs were taken.

### Vector design and assembly for gene deletion

Plasmids for gene replacement (Supplementary Fig. 3) were assembled by PCR with 50 ng of pUC19 plasmid, 160 ng of a hygromycin resistance cassette, 55 ng of upstream and 55 ng of downstream flanking region, and 10 μL of 2× HIFI DNA-assembling cloning solution (New England Biolabs, UK). Transformations of *E. coli* with each plasmid were performed using a Gibson cloning kit (New England Biolabs, UK). Colony PCR was performed to identify desired transformants using nested primers in the *hph* gene (Supplementary Table 4). Plasmid extractions were performed via a Miniprep system (Promega, UK). Plasmids were checked for correct assembly by restriction enzyme digests, which were also used to release deletion cassettes. Bands of the relevant size were excised and purified using a Monarch gel purification kit (New England Biolabs, UK).

### *Penicillium roqueforti* transformation protocol

A PEG-mediated protoplast transformation protocol for *P. roqueforti* was developed based on the procedure described for *A. niger*^46^. Mycelium of strain 74–88 was harvested and washed before treatment with Protoplast F (Megazyme, Ireland) until sufficient protoplasting had occurred. After transformation with 1–4 µg of selected cassettes, protoplasts were regenerated overnight prior to spreading of aliquots on PDA plates containing 20 µg/mL of hygromycin B and incubation at 28 °C until individual colonies appeared. See Cleere^31^ for full experimental details.

Individuals that formed colonies with non-standard pigmentation were selected for further investigation (Supplementary Fig. 14). To confirm deletion of the resident gene, PCR using primers designed against the target gene was performed together with a positive 74–88 gDNA control (Supplementary Table 4). Correct integration of the deletion cassette in the *P. roqueforti* genome was verified by positional PCR^45^ using primers located outside of the deletion cassette and compliments located in the *hph* gene (Supplementary Table 4).

### Complementation transformations of *Δalb1* and *Δabr2*

Gene deletion strains *Δalb1* and *Δabr2* were selected for complementation studies due to the importance of the polyketide synthase and laccase genes at the start and end of the melanin biosynthetic pathway. Transformation protocols were used as described above except that transformation cassettes contained a phleomycin resistance gene together with either the wild-type *alb1* or *abr2* gene for reinsertion, and selection plates contained phleomycin rather than hygromycin.

### Mycotoxin extraction and detection by UHPLC

Mycotoxins were extracted either from cultures on inducing agar medium or from cheese wheels that had been matured for 10–12 weeks. For agar extraction, a 100 µL spore aliquot containing 10^6^ spores was point inoculated onto 25 mL yeast extract sucrose (YES) agar in a 9 cm diameter Petri dish and grown for 6 days at 26 °C in the dark. Mycotoxin extraction and chromatographic detection was performed according to Houbraken et al.^48^ with some modifications. Six agar plugs along the colony diameter were taken and submerged in 800 µL of ethyl acetate/dichloromethane/methanol (3:2:1 v/v/v with 1% formic acid) and sonicated for 45 min using a Sanyo Soniprep 150 (Sanyo, UK). Extracts were dried in a Savant SPD121P SpeedVac Concentrator (Thermo Scientific) and re-dissolved in 400 µL methanol, sonicated for 10 min, and passed through a 0.2 µm filter (Minisart, Sartorius, UK) into amber vials and stored at −20 °C. For cheese studies, extractions were made using methods modified from Fontaine et al.^37^. Replicate 4 g batches of cheese were placed in 50 mL falcon tubes and 25 mL acetonitrile with 0.1% formic acid. Samples were homogenised for 4 min using a T-25 Ultraturrax (Janke and Kunkel, Germany). Hexane (20 mL) was added and tubes vortex mixed for 3 min before centrifugation for 10 min at 4000 rpm at 4 °C. A 10 mL aliquot of the acetonitrile phase was filtered (Grade 1 filter paper; Whatman, UK) and resulting samples dried by SpeedVac, prior to resuspension in 500 µL methanol. Extracts were finally passed through a 0.2 µm filter prior to storage in amber vials at −20 °C.

Chromatographic analysis for all extracts was performed using a UHPLC Dionex Ultimate 3000 (Thermo Scientific, UK) equipped with a diode array detector (DAD). Separation of 5 µL samples was achieved using an Accucore C_18_ column (150 × 2.1 mm, particle size 2.6 µm, Thermo Scientific, UK) with a 0.1% formic acid in water/acetonitrile (ACN) gradient at a flow rate 0.8 mL/min. The column temperature was held at 60 °C. Gradient conditions were: 15–65% ACN in 5 min, from 65–100% ACN in 1 min, hold at 100% ACN for 1 min, down to 15% ACN in 1 min, then 15% ACN for 1 min. Mycotoxins were identified by retention times and UV-VIS spectra compared to standards (Sigma, UK). Areas under the curve were used for metabolite quantification against standards.

### Detection of volatiles by SPME-GCMS

Solid phase microextraction (SPME) followed by GCMS analysis was used to assess any impact on production of flavour volatiles using methods adapted from Gkatzionis et al.^32^ and Price et al.^33^ Flasks (250 mL) containing 100 mL of UHT milk (Co-Op, UK) were inoculated with 10^7^ spores (in phosphate-buffered saline, Sigma, UK) with three biological replicates. Samples were incubated at 24 °C with continuous shaking (80 rpm) for 7 days. The contents were then homogenised using an Ultraturrax and 5 mL of the resulting slurry was transferred into 20 mL headspace vials (Thermo-Fisher, UK) which were stored at −20 °C. GCMS was performed on a TRACE 1310 gas chromatograph (Thermo-Fisher, UK) with extraction at 50 °C for 20 min with continuous shaking using a Stableflex 50/30 μm DVB/CAR/PDMS fibre and desorption at 230 °C for 0.1 min in splitless mode. Volatiles were separated on a TG-5MS column (length 30 m, I.D. 0.25 mm, film thickness 0.1 µm) using helium as carrier gas at constant pressure of 18 psi. An initial oven temperature of 40 °C was held for 2 min, then increasing at 6 °C/min to a final temperature 220 °C. Chromatography was coupled to an ISQ mass spectrometer (Thermo-Fisher, UK) with the transfer line held at 250 °C. Mass spectrometry was performed in positive ionisation mode at 70 eV, with an ion source temperature of 200 °C and detector scanning between 45–250 *m/z* at a scan rate of 2 scans/s. Volatile compounds were identified by comparing their retention times and mass spectra with those of the National Institute of Standards and Technology (NIST) library. Quantification was done based on extracted ion chromatograms (MS quantification). All data were processed using Chromeleon 7.2 software (Thermo-Fisher, UK). Principal component analysis and analysis of variance (ANOVA) were performed using Unscrambler 10 software (Camo, Norway) and GraphPad Prism 7.03, respectively. Elsewhere statistical analyses were made using Prism 9 for Mac (www.graphpad.com).

### Reporting summary

Further information on research design is available in the Nature Research Reporting Summary linked to this article.

### Supplementary information

Supplementary Information

Reporting Summary

## Data Availability

Gene sequences generated in this study have been deposited at GenBank (https://www.ncbi.nlm.nih.gov/genbank/; accession numbers OQ680622-OQ680627). All other data supporting the findings of this study are available in the paper and supplementary information.
