# Supplementary material for: New colours for old in the blue-cheese fungus Penicillium roqueforti
Source: NPJ Sci Food. 2024 Jan 8;8:3. doi: 10.1038/s41538-023-00244-9 (PMC10774375; doi:10.1038/s41538-023-00244-9)
Supplement: Supplementary file 1 — Supplementary Information [file 41538_2023_244_MOESM1_ESM.pdf]

Guide for Supplementary Information for Cleere MC et al. (NPJ Science of Food)

Supplementary Table 1 – Table showing transformation rates for DHN-melanin biosynthetic genes in *Penicillium roqueforti*.

Supplementary Table 2 – Table showing DNA mutations in UV-induced mutants and arising colony colours of *Penicillium roqueforti*.

Supplementary Table 3 – Table showing production of volatile compounds by parental versus melanin gene deletant strains of *Penicillium roqueforti*.

Supplementary Table 4 – Table showing list of primers used in the study and their specific applications.

Supplementary Figure 1 – Figure showing homologues of *Aspergillus fumigatus* DHN-melanin pathway genes identified in the *Penicillium roqueforti* FM164 genome through BLASTP searches.

Supplementary Figure 2 – Figure showing effect of enzyme inhibitors on colony colouration *of Penicillium roqueforti* isolate 74-88.

Supplementary Figure 3 – Figure showing transformation approach used for *Penicillium roqueforti*.

Supplementary Figure 4 – Figure showing agarose gels used to resolve products from positional PCR of gene transformants to assess correct cassette integration and gene replacement in *Penicillium roqueforti*.

Supplementary Figure 5 – Figure showing effect of gene complementation on restoring phenotype in *Penicillium roqueforti*.

Supplementary Figure 6 – Figure showing agarose gels used to resolve products from positional PCR of gene transformants to assess correct cassette integration in complementation strains of *Penicillium roqueforti*.

Supplementary Figure 7 – Figure showing wild type and representative UV colour mutant strains of *Penicillium roqueforti.*

Supplementary Figure 8 – Figure showing comparison of *Penicillium roqueforti* colony phenotypes between gene deletant transformation strains and UV mutants with the same effected gene.

Supplementary Figure 9 – Figure showing impact of enzyme inhibitors on colouration of colonies of *Penicillium roqueforti* UV colour mutants.

Supplementary Figure 10 – Figure showing PCA plot illustrating differences in profiles of production of volatile compounds between parental B20 and UV-induced colour mutant strains.

Supplementary Figure 11 – Figure showing PCA plot illustrating in profiles of production of volatile compounds between parental strain A22 and UV-induced colour mutant strains.

Supplementary Figure 12 – Figure showing percentage survival of spores (relative to non-UV exposed controls) of either *Penicillium roqueforti* parental isolate 74-88 or various DHN-melanin biosynthesis gene deletion strains following exposure to UV stress.

Supplementary Figure 13 – Figure showing dose response graph of UV-treated spores of *Penicillium roqueforti.*

Supplementary Figure 14 – Figure showing transformants of *Penicillium roqueforti* on selection plate containing hygromycin-B.

Supplementary Table 1. Transformation of all six targeted genes in the DHN-melanin biosynthetic pathway of *Penicillium roqueforti.* Success rates expressed as percentage of transformant colonies containing the correct integrating (replacement) selection cassette.

Batch	Protoplasts Produced	Protoplasts per Transformation	Target Genes	Vector Used per Transformation (µg)	Success Rate (%)
**One**	$4.0x10^6$	$8.0x10^5$	*alb1, ayg1, arp1, abr1*	1.0 - 1.5	11.5
**Two**	$3.6x10^7$	$5.0x10^6$	*alb1, ayg1, arp1, arp2, abr1, abr2*	1.1 - 2.2	5
**Three**	$1.3x10^7$	$3.1x10^6$	*arp2*	2.1 - 3.0	12.7
**Four**	$1.1x10^7$; $1.3x10^7$	$2.8x10^6$; $3.2x10^6$	*abr2*	3.4 - 5.3; 3.2 - 4.0	4.8

Supplementary Table 2. UV induced mutations in *Penicillium roqueforti*. Wild type parental isolate 74-88 and ascospore strain B20 produced both phenotypic (colour) and genotypic (gene & mutation) alterations. * indicates stop codon. 'None' indicates no coding change found in any of the DHN-melanin biosynthetic pathway genes.

Strain	Colour	Gene	Mutation (nucleic acid)	Mutation (amino acid)
**74-88-1**	Reddish-brown	*arp2*	GTT to GAT	V to D
**74-88-2**	Olive brown	*abr1*	ATG to GAA	YF to *N
**74-88-4**	Greyish-green	None	None	None
**74-88-5**	Green	*ayg1*	CTT to CCT	L to P
**74-88-6**	White (albino)	None	None	None
**74-88-10**	Intense blue	None	None	None
**74-88-12**	Mustard Fawn	*ayg1*	TGC to CGC	C to R
**A22-26**	Powder blue	None	None	None
**B20-1**	White (albino)	*alb1*	TTG to TCG	L to S

Supplementary Table 3. Production of volatile compounds by *Penicillium roqueforti* parental isolate 74-88 compared to various gene deletant strains in the melanin biosynthesis pathway (*Δalb1*, *Δayg1*, *Δarp1*, *Δarp2* and *Δabr2*). Aroma profiles as determined by SPME-GCMS analysis. P-values derived from one-way ANOVA showing significant ($p<0.05$) or non-significant (ns) ($p>0.05$) differences between 74-88 and the respective gene deletant strain. There were significant changes in levels of between 8-15 of the 26 flavour compounds compared to strain 74-88.

	**74-88 vs. *Δalb1***	**74-88 vs. *Δayg1***	**74-88 vs. *Δarp2***	**74-88 vs. *Δarp1***	**74-88 vs. *Δabr1***	**74-88 vs. *Δabr2***
**2-Propanone-1-methoxy**	0.0001	0.0001	0.0001	0.0001	0.0001	0.0001
**Butanal-3-methyl**	ns	ns	ns	ns	ns	ns
**Butanal-2-methyl**	ns	ns	ns	ns	ns	ns
**2-Pentanone**	ns	ns	ns	ns	ns	ns
**2-Pentanol**	0.0014	0.0002	0.0086	0.0019	0.0037	0.003
**1-Butanol-3-methyl**	ns	0.0016	ns	ns	ns	ns
**Butanoic acid**	0.0032	0.0379	ns	ns	ns	ns
**2-Hexanone**	ns	ns	ns	ns	ns	ns
**Butanoic acid ethyl ester**	0.0069	0.0088	0.0137	0.0074	0.0292	0.0111
**Butanoic acid-3-methyl**	ns	ns	ns	ns	ns	ns
**2-Heptanone**	ns	ns	ns	ns	ns	ns
**2-Heptanol**	0.0016	0.0003	0.005	0.001	ns	0.0065
**2-Heptanone-3-methyl**	0.0001	0.0194	0.0013	0.0014	ns	0.0197
**Butanoic acid 2-methylpropyl ester**	ns	ns	ns	ns	0.0061	ns
**Hexanoic acid**	0.0002	0.0113	0.0021	0.0074	0.0012	ns
**3-Octanone**	ns	0.0409	ns	ns	ns	ns
**Hexanoic acid ethyl ester**	0.0003	0.0008	0.0006	0.0003	0.005	0.0006
**Benzene 1-methoxy-4-methyl**	ns	ns	ns	ns	ns	ns
**Benzeneacetaldehyde**	ns	ns	ns	ns	ns	ns
**Butanoic acid 3-methylbutyl ester**	ns	0.0002	ns	ns	ns	ns
**8-Nonen-2-one**	ns	ns	ns	ns	ns	ns
**2-Nonanone**	ns	ns	0.0475	ns	0.0468	ns
**2-Nonanol**	0.002	0.0059	0.0012	0.0251	0.0024	0.0127
**Phenylethyl Alcohol**	ns	0.0064	ns	ns	ns	ns
**Isopentyl hexanoate**	ns	0.0148	ns	ns	ns	ns
**2-Undecanone**	0.002	0.0069	0.0003	ns	0.0002	0.0062

Supplementary Table 4. Master list of primers used in the present study specifying particular applications.

Sequencing Primers			
**Primer Set**	**Oligo Name**	**Sequence 5' to 3' (include modification codes if applicable)**	**Comments**
**1**	MCayg1aF	CCTTACCCTCGTCATCTTGG	Starts at 37
	MCayg1aR	TTGATGATCTCGTGGGAGCG	
**2**	MCayg1bF	AAGCAGGTGTACCTGAAAGC	Starts at 837
	MCayg1bR	GGCTGCGACTCTTTAAAGCT	Starts at 1926
**3**	arp2F	TCAACACGATTCCACACCCA	
	arp2R	TCAGATGGTAATTGGCCCTGA	
**4**	MCarp1F	GAAATATGCTGGCCAGATCC	Starts at 10
	MCarp1R	GTTGTACCAGTTTCCCTTCG	Starts at 779
**5**	MCarb1aF	AGCATGGCAGTGGAGTTATG	Starts at 6
	MCarb1aR	CTGCAGAACCTGGAATTGGT	Starts at 1090
**6**	MCarb1bF	ATCGCTCTACACTGCCAACA	Starts at 916
	MCarb1bR	AGACTTGCGGGTATTATCGG	Starts at 2029
**7**	MCalb1aF	CCTTAAGTTTTCGCCTGACC	Starts at 22
	MCalb1aR	CTTGCCTGTCTCACTTGACA	Starts at 1077
**8**	alb1bF	GTTCCAATCCACGGCCCTTA	
	alb1bR	AACCGACTGCATCTCCACTG	
**8/9 Gap**	MCalb1b/cF	TAGTGTGGATACCGCTTGCT	Starts at 1848
	MCalb1b/cR	CACATTCTCGTCATTGAGCG	Starts at 2667
**9**	alb1cF	TCCAACAGCGACCCAAAAGA	
	alb1cR	GCTGGGCGCATTGATACAAG	
**10**	alb1dF	ACACAATCTACCTTGCCGGG	
	alb1dR	GATGAGGTAAGAGCTGCGCT	
**10/11 Gap**	MCalb1d/eF	GCTGCGCCAGTTTCTGTTTT	Starts at 4066
	MCalb1d/eR	ACGCTCGGATCGAACTTCTT	Starts at 4929
**11**	MCalb1eF	GAAGAAGATCCTTGACCACG	Starts at 4495
	MCalb1eR	TGCTGTCATTTTGCTCCTGC	Starts at 5629
**11/12 Gap**	MCalb1e/fF	TTCCAAGCTCTGGCACGAAA	Starts at 5097
	MCalb1e/fR	ACGCCGAATCTCAGCAATGT	Starts at 6155
**12**	alb1fF	TCGAGTCCAAACAGCAGTGG	
	alb1fR	ATACGTTTACGCCGAAGCCA	
**13**	arb2aF	AGGAAGGCGGTAAGAAAGGC	
	arb2aR	GGGCAAATTGTTGGTCACGT	
**14**	MCarb2bF	GTCGAGAGGGAGATGATCTT	Starts at 926
	MCarb2bR	GCAGGTAGGATAGCAGGATA	Starts 2280
**15**	arb2cF	GCGCTACTCTGCCATGATCA	
	arb2cR	CGATGATGATGCCAGTCCCA	
**Mix**	Alb1mixF	AAGGTGCTCAGTACACAGGC	
	Alb1mixR	CGCCCTTTCCAATTTCAGCC	
**Transformation Primers**			
**Alb1**			
**Primer Set**	**Oligo Name**	**Sequence 5' to 3' (include modification codes if applicable)**	**Comments**
**Upstream**	Upstream_Alb1 Fwd	ACCTGCAGGCATGCAAGCTTCAGATGATGCCTGCAGATG	PUC19
	Upstream_Alb1 Rev	GCAGAGCTAATCTTCCTGGTAGCAGATTAATG	hph
**hph**	hph_Alb1 Fwd	AGGAAGATTAGCTCTGCAAAGGGCGTT	Upstream Alb1

	hph_Alb1 Rev	GCAATACGTCAGTGTGATGGAATTCGC	Downstream Alb1
**Downstream**	Downstream_Alb1 Fwd	TCACACTGACGTATTGCATTCGGGGAC	hph
	Downstream_Alb1 Rev	GACCATGATTACGCCAAGCTTACCGAAAACCGCCATTTG	PUC19
		**Ayg1**	
**Primer Set**	**Oligo Name**	**Sequence 5' to 3' (include modification codes if applicable)**	**Comments**
**Upstream**	Upstream_Ayg1 Fwd	AATTCGAGCTCGGTACCACTCGCTGCTTTGCTGCT	PUC19
	Upstream_Ayg1 Rev	GCAGAGCTTTTGAGCGGGATATAGACG	hph
**hph**	hph_Ayg1 Fwd	CGCTCAAAAGCTCTGCAAAGGGCGTT	Upstream Ayg1
	hph_Ayg1 Rev	GCACCCACCAGTGTGATGGAATTCGC	Downstream Ayg1
**Downstream**	Downstream_Ayg1 Fwd	TCACACTGGTGGGTGCTGTGTCAATATAG	hph
	Downstream_Ayg1 Rev	AGGATCCCCGGGTACCGAATTCAGGAAGCTGAAGATG	PUC19
		**Arp2**	
**Primer Set**	**Oligo Name**	**Sequence 5' to 3' (include modification codes if applicable)**	**Comments**
**Upstream**	Upstream_Arp2 Fwd	AATTCGAGCTCGGTACCATGAGAGTGACCACGAAGC	PUC19
	Upstream_Arp2 Rev	GCAGAGCTTTTGGCGGTTATTTTGATG	hph
**hph**	hph_Arp2 Fwd	CCGCCAAAAGCTCTGCAAAGGGCGTT	Upstream Arp2
	hph_Arp2 Rev	GACATCGACAGTGTGATGGAATTCGC	Downstream Arp2
**Downstream**	Downstream_Arp2 Fwd	TCACACTGTCGATGTCTCGTGGGGTTGCC	hph
	Downstream_Arp2 Rev	AGGATCCCCGGGTACCGGCGCGGTCGAGATGGCC	PUC19
		**Arp1**	
**Primer Set**	**Oligo Name**	**Sequence 5' to 3' (include modification codes if applicable)**	**Comments**
**Upstream**	Upstream_Arp1 Fwd	AATTCGAGCTCGGTACCTCTTGCTGCCCGCATAC	PUC19
	Upstream_Arp1 Rev	GCAGAGCTGGTTGTTCGGCAATCTTTG	hph
**hph**	hph_Arp1 Fwd	GAACAACCAGCTCTGCAAAGGGCGTT	Upstream Arp1
	hph_Arp1 Rev	CATTGACATCAGTGTGATGGAATTCGC	Downstream Arp1
**Downstream**	Downstream_Arp1 Fwd	TCACACTGATGTCAATGGGCTTTTGC	hph
	Downstream_Arp1 Rev	AGGATCCCCGGGTACCTTAAATTATCTGAAAGAAGTGTCG	PUC19
		**Abr1**	
**Primer Set**	**Oligo Name**	**Sequence 5' to 3' (include modification codes if applicable)**	**Comments**
**Upstream**	Upstream_Abr1 Fwd	AATTCGAGCTCGGTACCGACTATTCTTCTTCATCATG	PUC19
	Upstream_Abr1 Rev	GCAGAGCTATGTTCATCTTTTCCACCG	hph
**hph**	hph_Abr1 Fwd	GATGAACATAGCTCTGCAAAGGGCGTT	Upstream Abr1
	hph_Abr1 Rev	TGCTGGCACAGTGTGATGGAATTCGC	Downstream Abr1
**Downstream**	Downstream_Abr1 Fwd	TCACACTGTGCCAGCACCTGTGATATG	hph
	Downstream_Abr1 Rev	AGGATCCCCGGGTACCATCCCCACTCAGAGTCGTATC	PUC19
		**Abr2**	
**Primer Set**	**Oligo Name**	**Sequence 5' to 3' (include modification codes if applicable)**	**Comments**

**Upstream**	Upstream_Abr2 Fwd	AATTCGAGCTCGGTACCACTAGGGTGCAAATACTCATG	PUC19
	Upstream_Abr2 Rev	GCAGAGCTTTTGTGGAGTTGGTTGGAG	hph
**hph**	hph_Abr2 Fwd	CTCCACAAAAGCTCTGCAAAGGGCGTT	Upstream Abr2
	hph_Abr2 Rev	TAGAACACACAGTGTGATGGAATTCGC	Downstream Abr2
**Downstream**	Downstream_Abr2 Fwd	TCACACTGTGTGTTCTATGATTTTTCTATCC	hph
	Downstream_Abr2 Rev	AGGATCCCCGGGTACCATCAACGGGTATGCGATC	PUC19
**Universal Hygromycin Primers**			
**Primer Set**	**Oligo Name**	**Sequence 5' to 3' (include modification codes if applicable)**	**Comments**
**hph**	Forward	AGCTCTGCAAAGGGCGTT	
	Reverse	CAGTGTGATGGAATTCGCCCTT	
**Hygromycin Colony PCR Primers**			
**Primer Set**	**Oligo Name**	**Sequence 5' to 3' (include modification codes if applicable)**	**Comments**
**hph**	hph_for	CGATGTAGGAGGGCGTGGATA	
	hph_rev	GCTTCTGCGGGCGATTTGTGT	
**Positional PCR Primers**			
**Primer Set**	**Oligo Name**	**Sequence 5' to 3' (include modification codes if applicable)**	**Comments**
**Abr1**	Abr1-FF	TTCAGTCCCATCATCAGGAGCC	
	Abr1-FR	ATCTCCAGTCAATCTCCCGC	
**Abr2**	Abr2-Ff	ACCTCTACCATTGACCGCTC	
	Abr2-FR	ACTCGGTGCCTGGTTCATTG	
**Alb1**	Alb1-FF	CGGCAGATCTTATTCCGTCG	
	Alb1-FR	CCATGTAACTCGCCAAGAGC	
**Ayg1**	Ayg1-FF	CCATCAACAGCATTTATCGTCC	
	Ayg1-FR	TTGTCCGAGACTACACTGGAGG	
**Arp1**	Arp1-FF	TTGGCTCCACCGTTACAAGG	
	Arp1-FR	TGTCCCGTTTTGCGTCCAAG	
**Arp2**	Arp2-FF	AGCCGCCTCACTATGGAAAC	
	Arp2-FR	GGCAGAGAAGAAGGTGTATCTATC	
**Hph1**	Hph1-FF	ACAATGCCTGAACTCACCGC	
	Hph1-FR	TCTACAAATGGTGGAGGCGG	
**Pos/hph**	Hph2-FF	GCATTAATGCATTGGACCTCG	
	Hph2-FR	GCTGAAGTCGATTTGAGTCC	
**Complementation Phleomycin Primers**			
**Primer Set**	**Oligo Name**	**Sequence 5' to 3' (include modification codes if applicable)**	**Comments**
**Alb1**	PUC19	CGACGTTGTAAAACGACGGCCAGTGAATTCAGATGATGCCTGCAGATG	5_Flank
	Alb1_gene	TGGGGCCCTCCATAATCTTCCTGGTAGCAGATTAATG	5_Flank
	5_Flank	TACCAGGAAGATTATGGAGGGCCCCAGTCAT	Alb1_gene
	Phle	TTGTGGTGAGCGGCCTTACGCCGAAGCCATAGAG	Alb1_gene
	Alb1_gene	GGCTTCGGCGTAAGGCCGCTCACCACAAAAG	Phle
	3_Flank	GAATGCAATACGTGCGAGTGAGGGTTGAGTACG	Phle
	Phle	AACCCTCACTCGCACGTATTGCATTCGGGGA	3_Flank
	PUC19	AGAGGATCCCCGGGTACCGAGCTCGAATTCCTACAAAGGAAGGAAGAAGAG	3_Flank
**Abr2**	PUC19	AGTGAATTCGAGCTCGGTACCCGGGGATCCACTAGGGTGCAAATACTCATG	5_Flank
	Abr2_gene	TCGTGTGTGTCATTTTGTGGAGTTGGTTGGAG	5_Flank
	5_Flank	CCAACTCCACAAAATGACACACACGAAGTCC	Abr2_gene
	Phle	TTGTGGTGAGCGGCCCTATAGGTAGTGAGGAGGAACTTC	Abr2_gene
	Abr2_gene	TCACTACCTATAGGGCCGCTCACCACAAAAG	Phle
	3_Flank	ATCATAGAACACAGCGAGTGAGGGTTGAGTACG	Phle
	Phle	AACCCTCACTCGCTGTGTTCTATGATTTTTCTATCC	3_Flank
	PUC19	GCATGCCTGCAGGTCGACTCTAGAGGATCCGTATATACCTTTCTTACTTAATGGTTTC	3_Flank

Heterologous Expression Primers			
Primer Set	Oligo Name	Sequence 5' to 3' (include modification codes if applicable)	Comments
PksP	tagX_Proq_ PksP_fw	ATCACCATCACCATGGAATGGAGGGCCCCAGTCATTTG	
	Proq_PksP_ tag_rv	CTGCTGTTATCCATGGTTACGCCGAAGCCATAGAGTTAG	
Ayg1	IF_Ayg1_ TagSMx_f	GCATCATCATCACCATCACCATGGAGTGAATTGGATCCTCGGA	
	IF_Ayg1_ TagSMx_rv	TTGAAATCACTGCTGTTATCCATGGTCAGTTCTTCACCTCCTTC	

Alb1

Conidial yellow pigment biosynthesis polyketide synthase [Penicillium roqueforti FM164]

GenBank: CDM29654.1

Identical Proteins FASTA Graphics

Go to:

```
LOCUS       CDM29654                2136 aa            linear   PLN 06-JUL-2015
DEFINITION  Conidial yellow pigment biosynthesis polyketide synthase
            [Penicillium roqueforti FM164].
ACCESSION   CDM29654
VERSION     CDM29654.1
DBLINK      BioProject: PRJEB4023
            BioSample: SAMEA3138836
DBSOURCE    embl accession HG792015.1
```

Ayg1

unnamed protein product [Penicillium roqueforti FM164]

GenBank: CDM29601.1

Identical Proteins FASTA Graphics

```
LOCUS       CDM29601                409 aa            linear   PLN 06-JUL-2015
DEFINITION  unnamed protein product [Penicillium roqueforti FM164].
ACCESSION   CDM29601
VERSION     CDM29601.1
DBLINK      BioProject: PRJEB4023
            BioSample: SAMEA3138836
DBSOURCE    embl accession HG792015.1
```

Arp1

Probable scytalone dehydratase [Penicillium roqueforti FM164]

GenBank: CDM29603.1

Identical Proteins FASTA Graphics

Go to:

```
LOCUS       CDM29603                171 aa            linear   PLN 06-JUL-2015
DEFINITION  Probable scytalone dehydratase [Penicillium roqueforti FM164].
ACCESSION   CDM29603
VERSION     CDM29603.1
DBLINK      BioProject: PRJEB4023
            BioSample: SAMEA3138836
DBSOURCE    embl accession HG792015.1
```

Arp2

Short-chain dehydrogenase/reductase SDR [Penicillium roqueforti FM164]

GenBank: CDM29602.1

Identical Proteins FASTA Graphics

Go to:

```
LOCUS       CDM29602                 268 aa            linear   PLN 06-JUL-2015
DEFINITION  Short-chain dehydrogenase/reductase SDR [Penicillium roqueforti
            FM164].
ACCESSION   CDM29602
VERSION     CDM29602.1
DBLINK      BioProject: PRJEB4023
            BioSample: SAMEA3138836
DBSOURCE    embl accession HG792015.1
```

Abr1

Cupredoxin [Penicillium roqueforti FM164]

GenBank: CDM29606.1

Identical Proteins FASTA Graphics

Go to:

```
LOCUS       CDM29606                 546 aa            linear   PLN 06-JUL-2015
DEFINITION  Cupredoxin [Penicillium roqueforti FM164].
ACCESSION   CDM29606
VERSION     CDM29606.1
DBLINK      BioProject: PRJEB4023
            BioSample: SAMEA3138836
DBSOURCE    embl accession HG792015.1
```

Abr2

Laccase-1 [Penicillium roqueforti FM164]

GenBank: CDM30362.1

Identical Proteins FASTA Graphics

Go to:

```
LOCUS       CDM30362                 586 aa            linear   PLN 06-JUL-2015
DEFINITION  Laccase-1 [Penicillium roqueforti FM164].
ACCESSION   CDM30362
VERSION     CDM30362.1
DBLINK      BioProject: PRJEB4023
            BioSample: SAMEA3138836
DBSOURCE    embl accession HG792016.1
```

Supplementary Figure 1. Homologues of *Aspergillus fumigatus* DHN-melanin pathway genes identified in the *Penicillium roqueforti* FM164 genome through BLASTP searches.

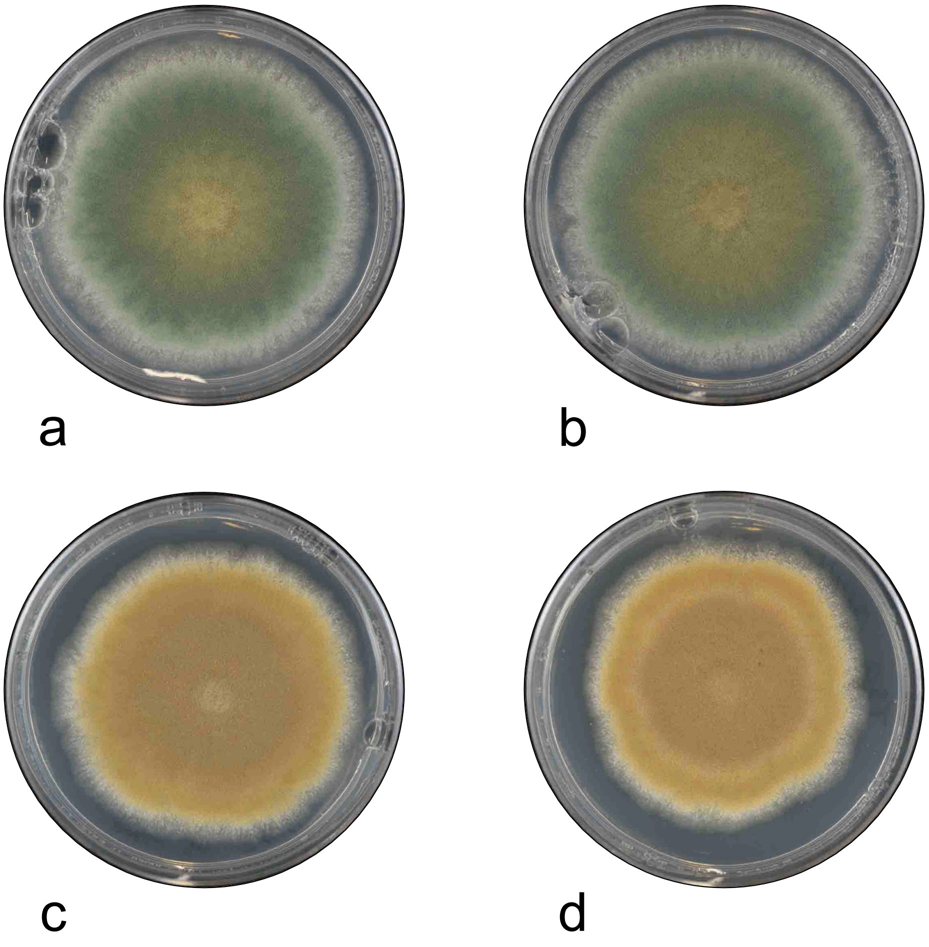

Supplementary Figure 2. **Effect of enzyme inhibitors on colony colouration *of Penicillium roqueforti* isolate 74-88. a** Non-supplemented medium, **b** Ethanol control, **c** Pyroquilon, **d** Tricyclazole. Compounds were added to the growth media at concentrations between 4-30 µg/mL.

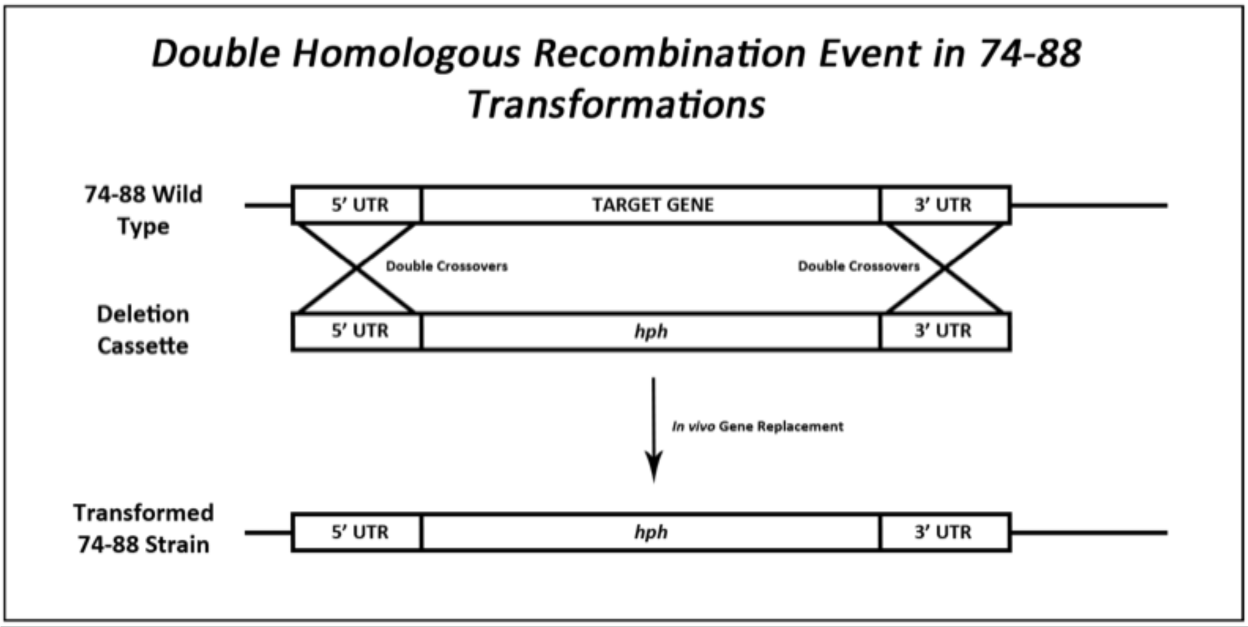

Supplementary Figure 3. *Penicillium roqueforti* transformation approach. Double homologous recombination events through 5' and 3' flanking regions replace the targeted pigment biosynthesis gene with a hygromycin resistance gene (*hph*) used for selection.

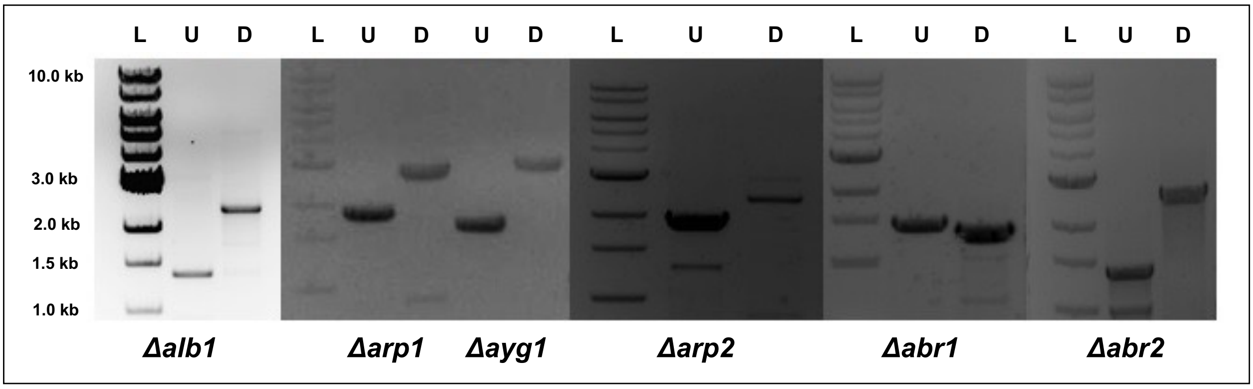

Supplementary Figure 4. Agarose gels used to resolve products from positional PCR of gene transformants to assess correct cassette integration and gene replacement. L = 1 kb ladder, U = upstream region, D = downstream region. Predicted sizes for correct integration: *Δalb1*U = 1427 bp, *Δalb1*D = 2500 bp; *Δarp1U* = 1883 bp, *Δarp1D* = 2679 bp; *Δayg1U* = 1749 bp, *Δayg1D* = 2761 bp; *Δarp2U* = 1784 bp, *Δarp2D* = 2548 bp; *Δabr1U* = 1480 bp *Δabr1D* = 1351 bp; *Δabr2*U = 1351 bp, *Δabr2*D = 2654 bp.

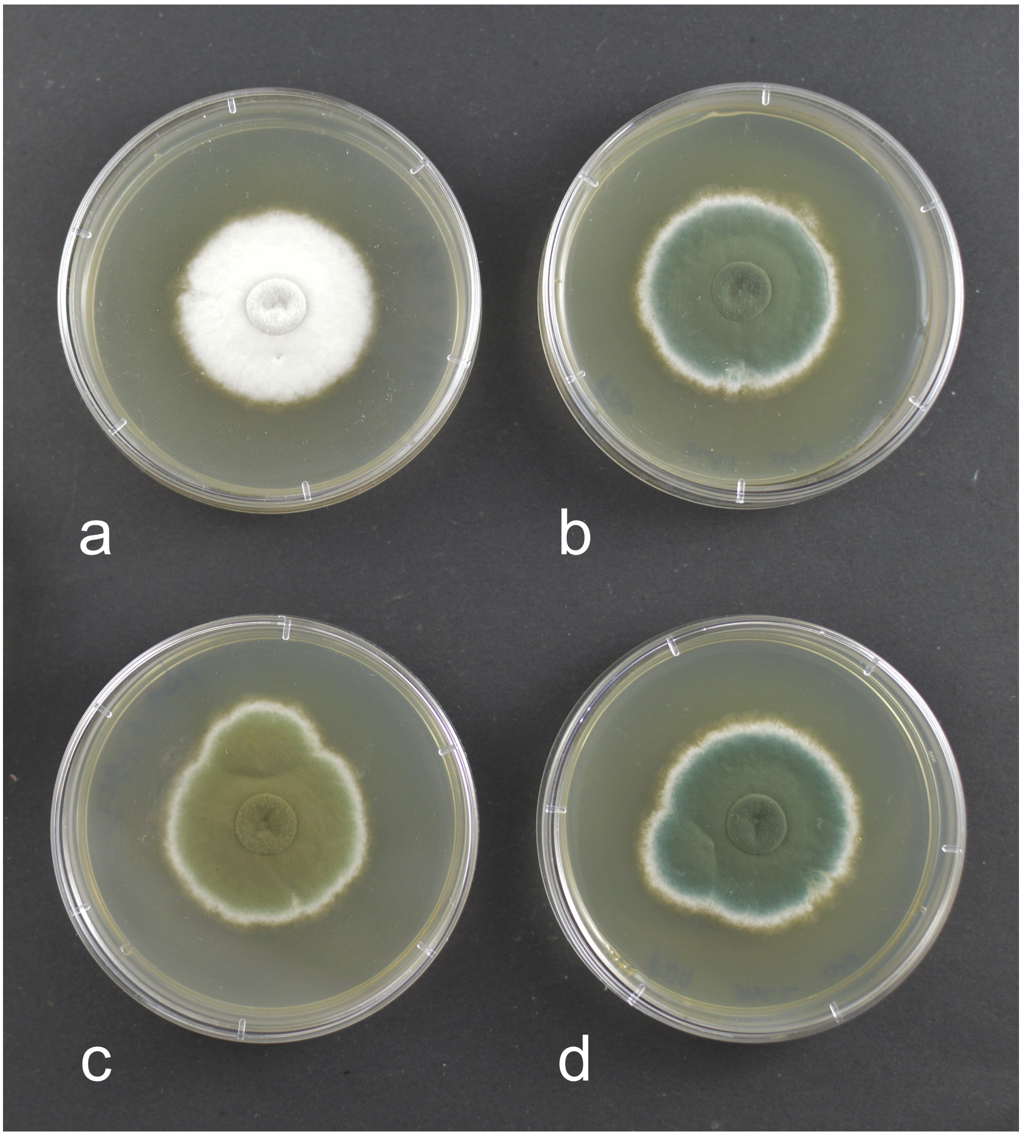

Supplementary Figure 5. Gene complementation. Experiments showing that transformation (re-integration) of the parental gene rescues and restores wild-type colour expression in colour mutants of *Penicillium roqueforti* isolate 74-88. Figure shows gene deletant strains **a** *Δalb1* and **c** *Δabr2*, as compared to gene complementation strains **b** c*Δalb1* and **d** c*Δabr2*.

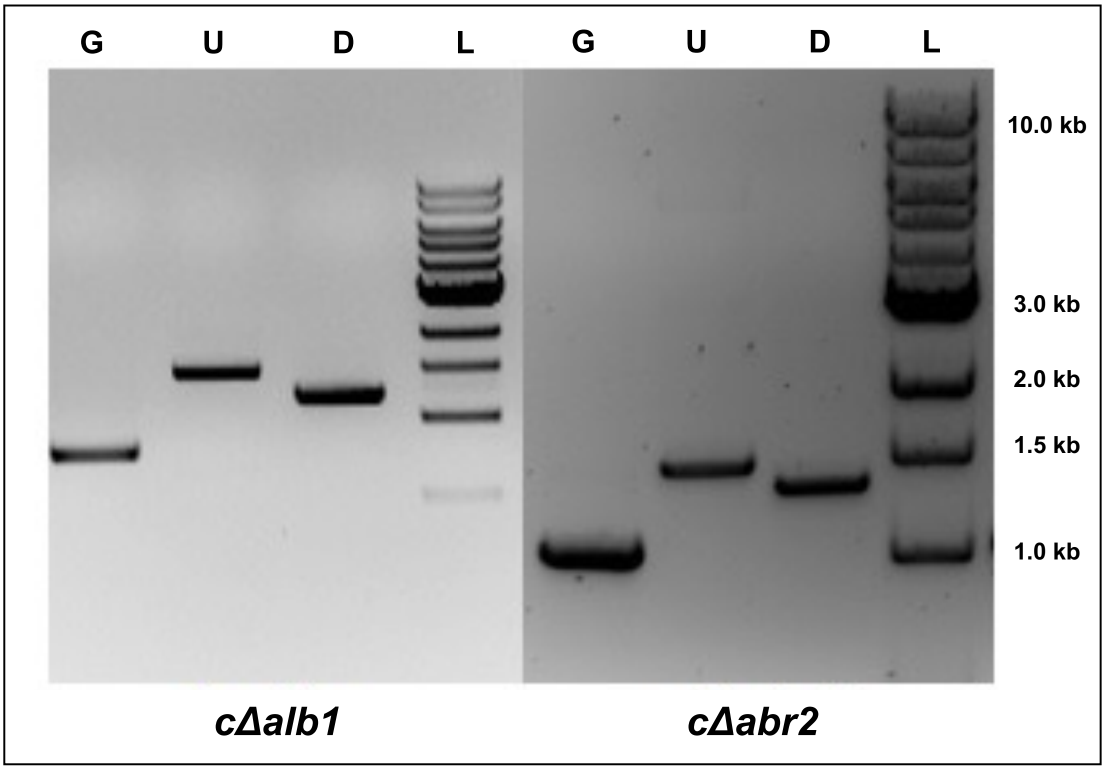

Supplementary Figure 6. Agarose gels used to resolve products from positional PCR of gene transformants to assess correct cassette integration in complementation gene strains. L = 1 kb ladder, U = upstream region, D = downstream region, G = Gene + Phleomycin nested PCR product. Predicted sizes for correct integration: *Δalb1*G = 787 bp, c*Δalb1*U = 1490 bp, c*Δalb1*D = 1166 bp; c*Δabr2*G = 1056 bp, c*Δabr2*U = 1635 bp, c*Δabr2*D = 1359 bp.

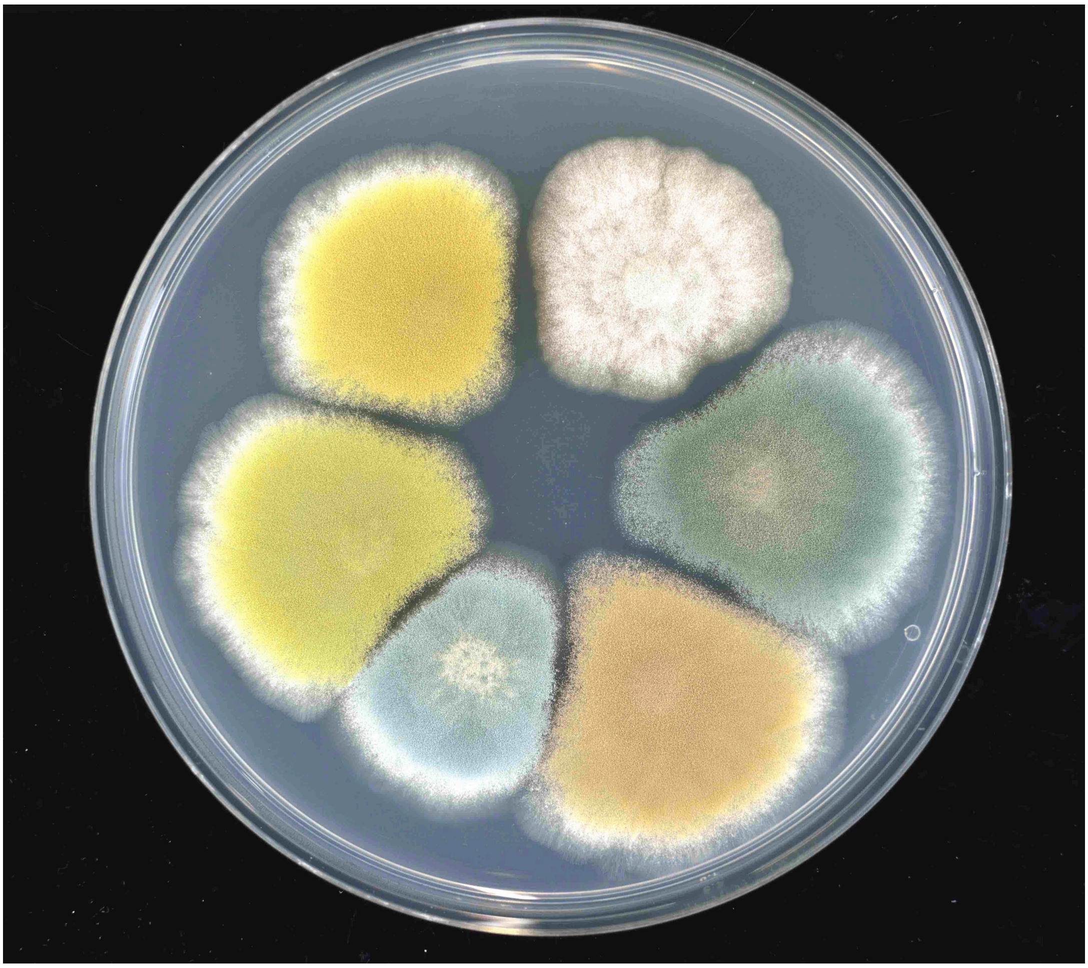

Supplementary Figure 7. **Wild type and representative UV colour mutant strains of *Pencillium roqueforti.*** Clockwise from top right strains are 74-88-6 (white), 74-88 (wild-type parent), 74-88-1 (reddish-brown), 74-88-10 (intense blue), 74-88-5 (green), and 74-88-12 (mustard fawn).

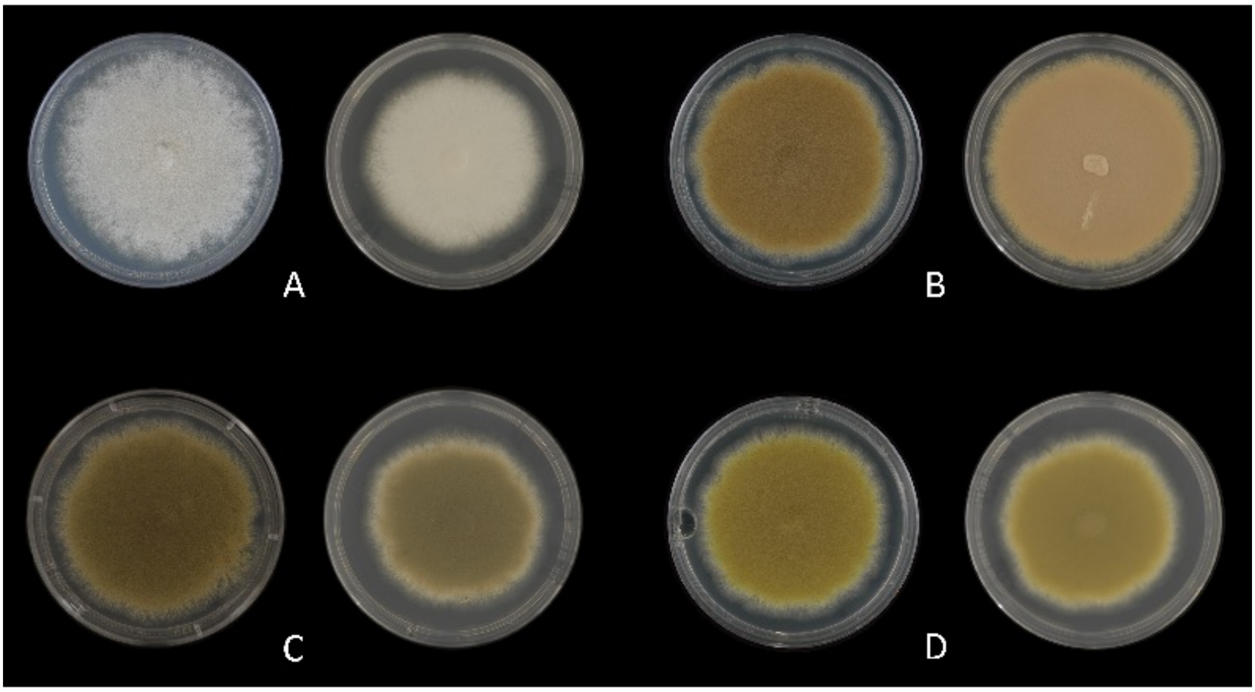

Supplementary Figure 8. Comparison of *Penicillium roqueforti* colony phenotypes between gene deletant transformation strains and UV mutants with the same effected gene. a B20-1 (*alb1*-L102S) [left] and 74-88 *Δalb1* [right]; **b** 74-88-1 (*arp2*-T290A) [left] and 74-88 *Δarp2* [right]; **c** 74-88-2 (*abr1*-Y42*;D43N) [left] and 74-88 *Δabr1* [right]; **d** 74-88-12 (*ayg1*-C344R) [left] and 74-88 *Δayg1* [right].

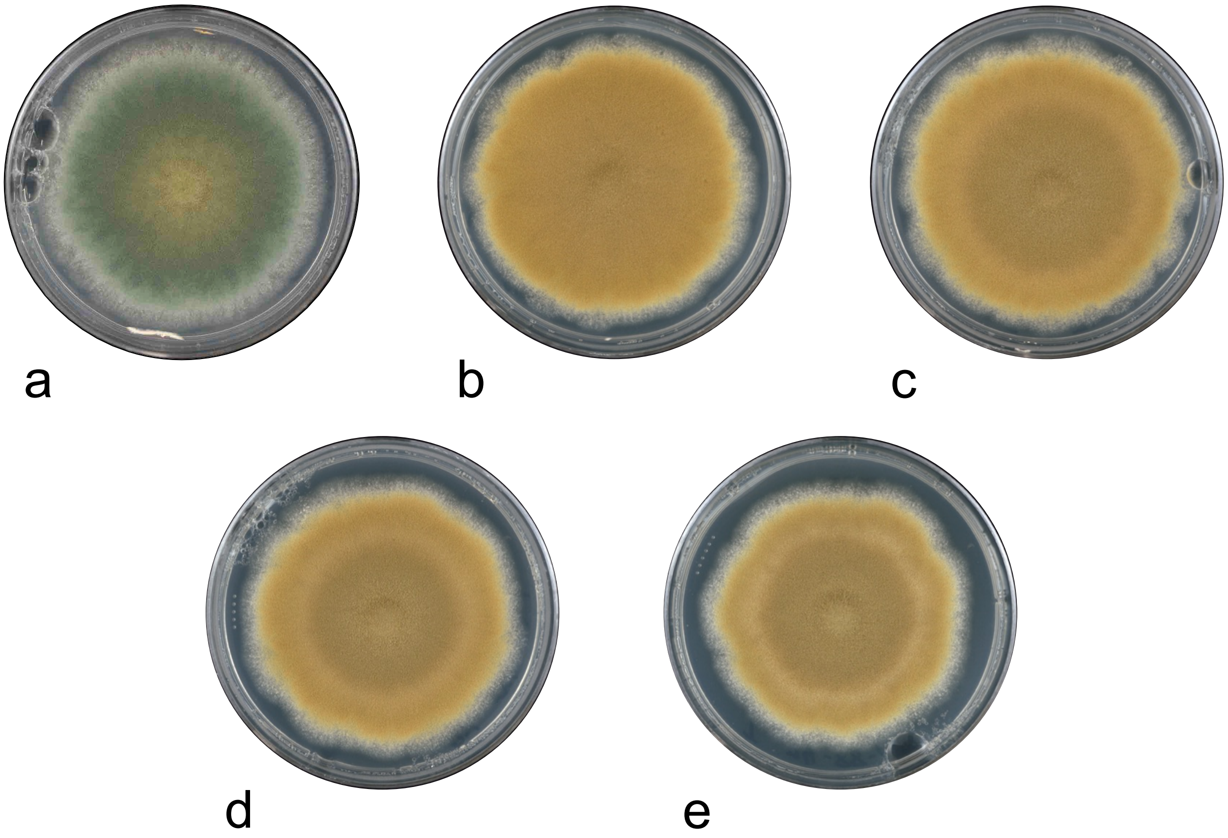

Supplementary Figure 9. Impact of enzyme inhibitors on colouration of colonies of *Penicillium roqueforti* UV colour mutant 74-88-1 (*arp2*-T290A). a 74-88 parent, **b** 74-88-1 on non-supplemented medium, **c** 74-88-1 on ethanol control medium, **d** 74-88-1 on Pyroquilon supplemented medium, **e** 74-88-1 on Tricyclazole supplemented medium. Compounds were added to the growth media to a final concentration of 30 µg/ml. Meanwhile, when colour mutants earlier in the putative DHN-melanin pathway were exposed to tricyclazole and pyroquilon, such as 74-88-12 (ayg1-C344R) that produced mustard fawn colonies, no change was seen in the colony colouration. This could be explained by the mutation in the ayg1 gene perturbing 1,3,6,8-THN production and causing any downstream enzyme inhibitors to have no effect (results not shown).

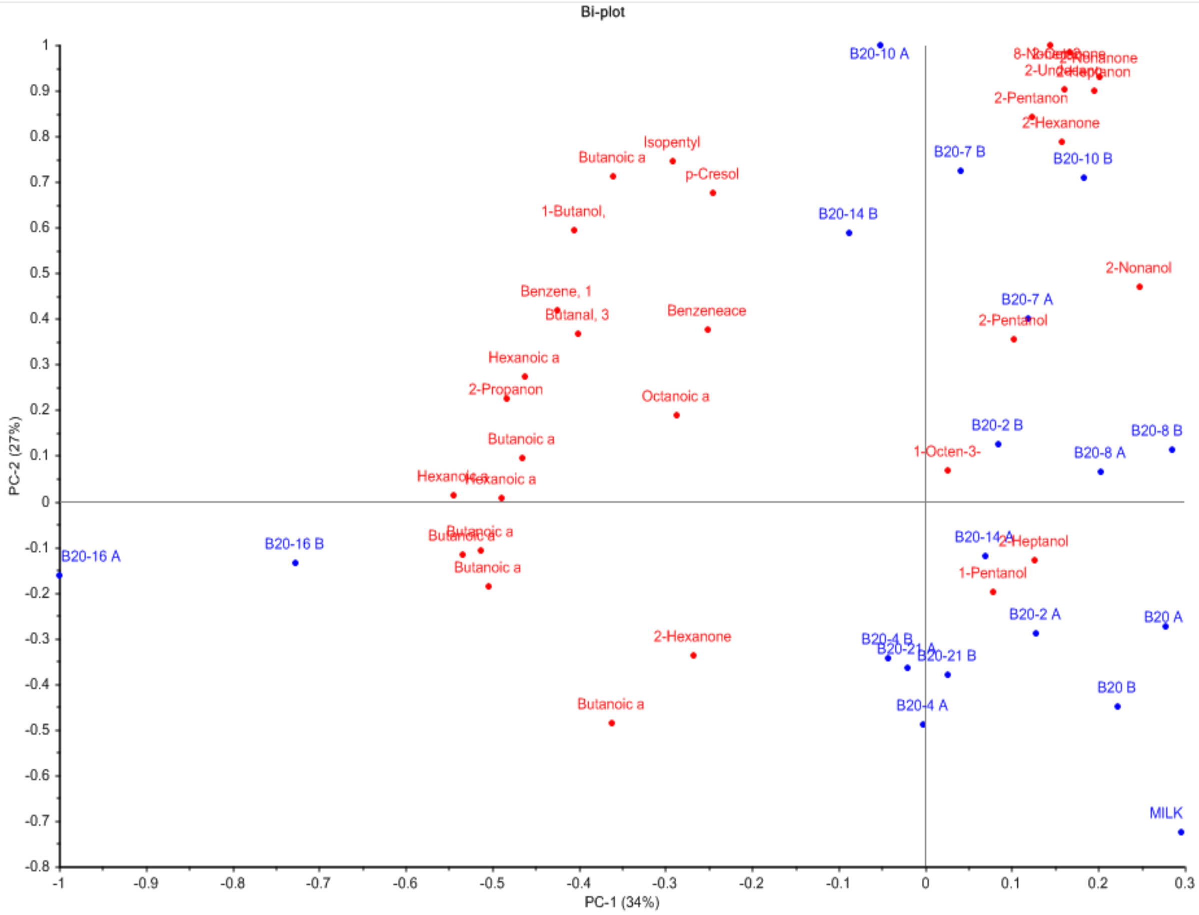

Supplementary Figure 10. PCA plot to show differences in profiles of production of volatile compounds (displayed in red) between the parental strain B20 and various UV-induced colour mutant strains. B20-2, B20-4, B20-7, B20-8, B20-10, B20-14, B20-16, and B20-21 displayed in blue. Note that suffixes (A) and (B) indicate replicate GC-MS trials.

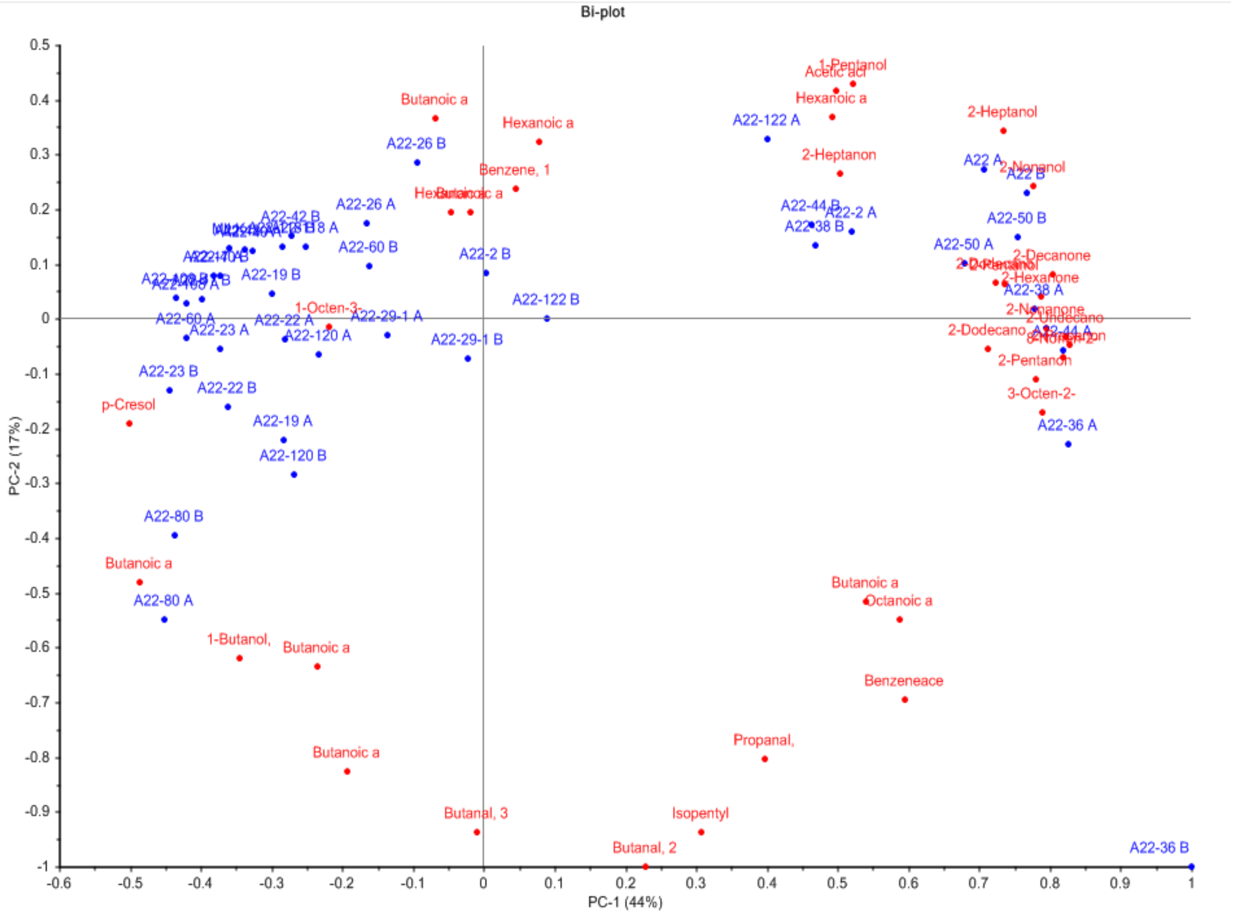

Supplementary Figure 11. PCA plot to show differences in profiles of production of volatile compounds (displayed in red) between the parental strain A22 and various UV-induced colour mutant strains. A22-2, A22-17, A22-19, A22-22, A22-23, A22-26, A22-29, A22-36, A22-38, A22-40, A22-42, A22-44, A22-50, A22-60, A22-80, A22-108, A22-118, A22-120, and A22-122 displayed in blue. Note that suffixes (A) and (B) indicate replicate GC-MS trials.

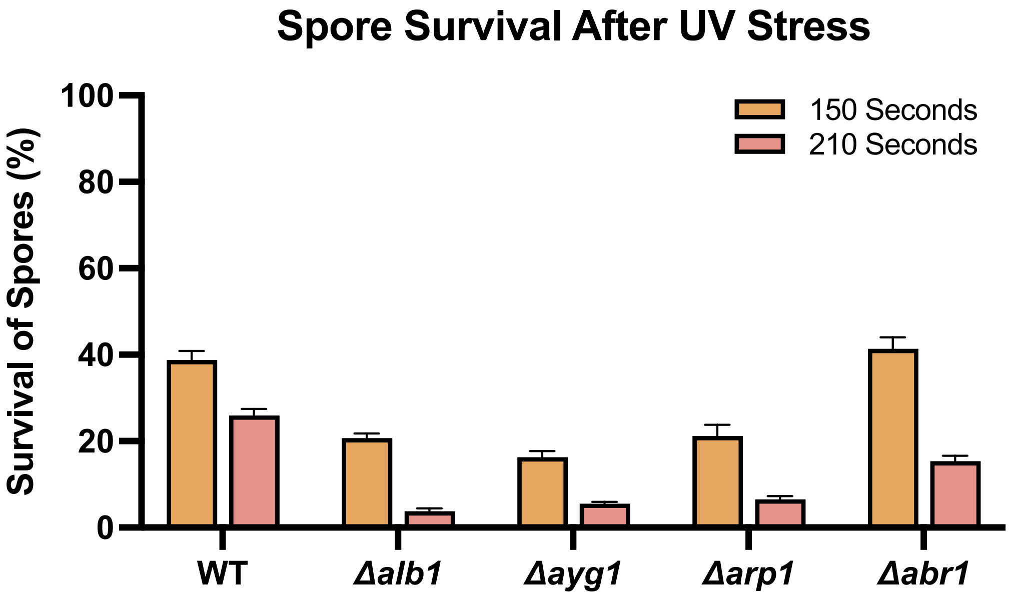

Supplementary Figure 12. Percentage survival of spores (relative to non-UV exposed controls) of either *Penicillium roqueforti* parental isolate 74-88 (WT) or various DHN-melanin biosynthesis gene deletion strains (*Δalb1, Δayg1, Δarp1,* and *Δabr1*) following exposure to UV stress for either 150 or 210 sec. For viability tests, 10 mL of a spore suspension of concentration 2,000/mL in 0.1% tween 20 was exposed in an open 5 cm diameter Petri dish to a Sylvania germicidal UV-C source emitting at a wavelength of 254 nm with an intensity of 100 μW cm^{-2}, whilst continuously stirring the spore suspension using a magnetic stirrer. There was a significant variation in spore survival between the various strains (two way ANOVA: F=48.4; DF=1,40; p<0.0001). Gene deletants showed up to an 8-fold decrease in percentage survival relative to 74-88 at the longest UV exposure time. Error bars indicate ±SEM.

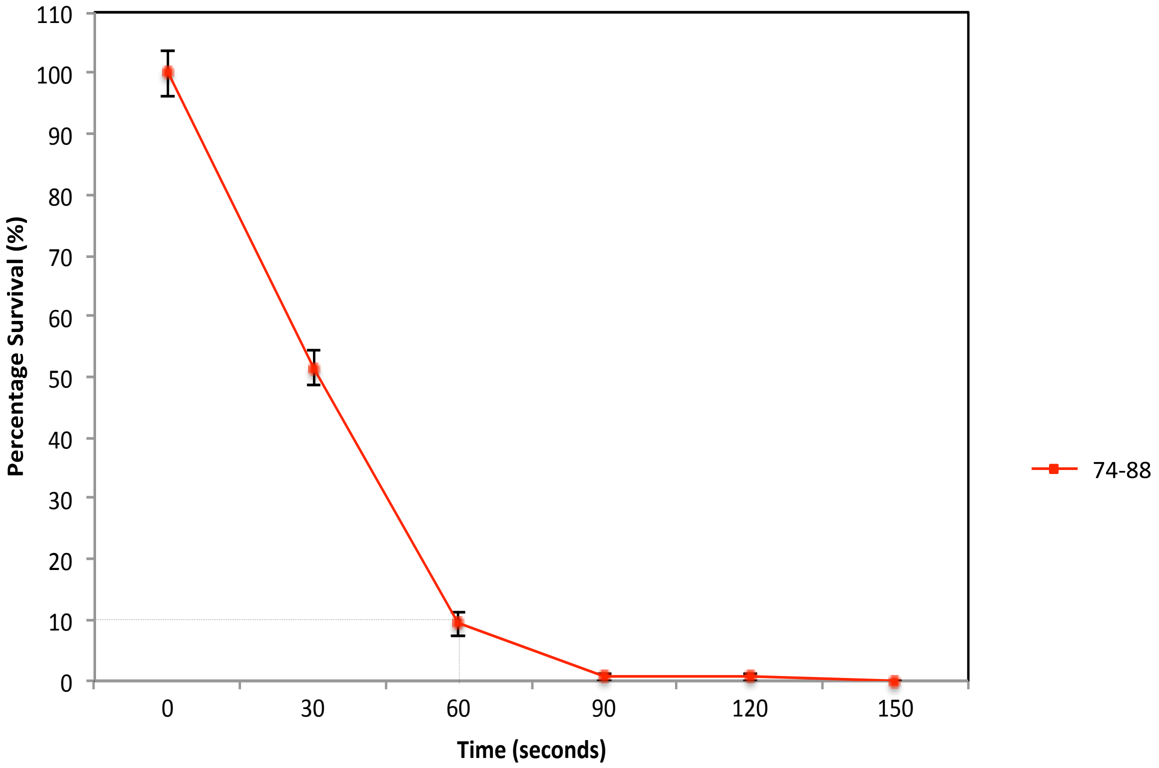

Supplementary Figure 13. UV dose response graph. Percentage survival of spores (relative to non-UV treated controls) dependent on time of exposure for conidia of *Penicillium roqueforti* isolate 74-88. Error bars indicate ±SEM. This involved exposure of 10 mL of a 200,000 spores/mL suspension in an open 5 cm diameter Petri dish for 60 sec to a Sylvania germicidal UV-C source emitting at a wavelength of 254 nm with an intensity of 100 μW cm^{-2} (corresponding to 0.0001 Joule s^{-1}), whilst mixing using a sterilised magnetic stirrer. The intensity of UV exposure was determined using a J-225 Blak-Ray UV meter from Ultra-Violet Products (San Gabriel, California, USA).

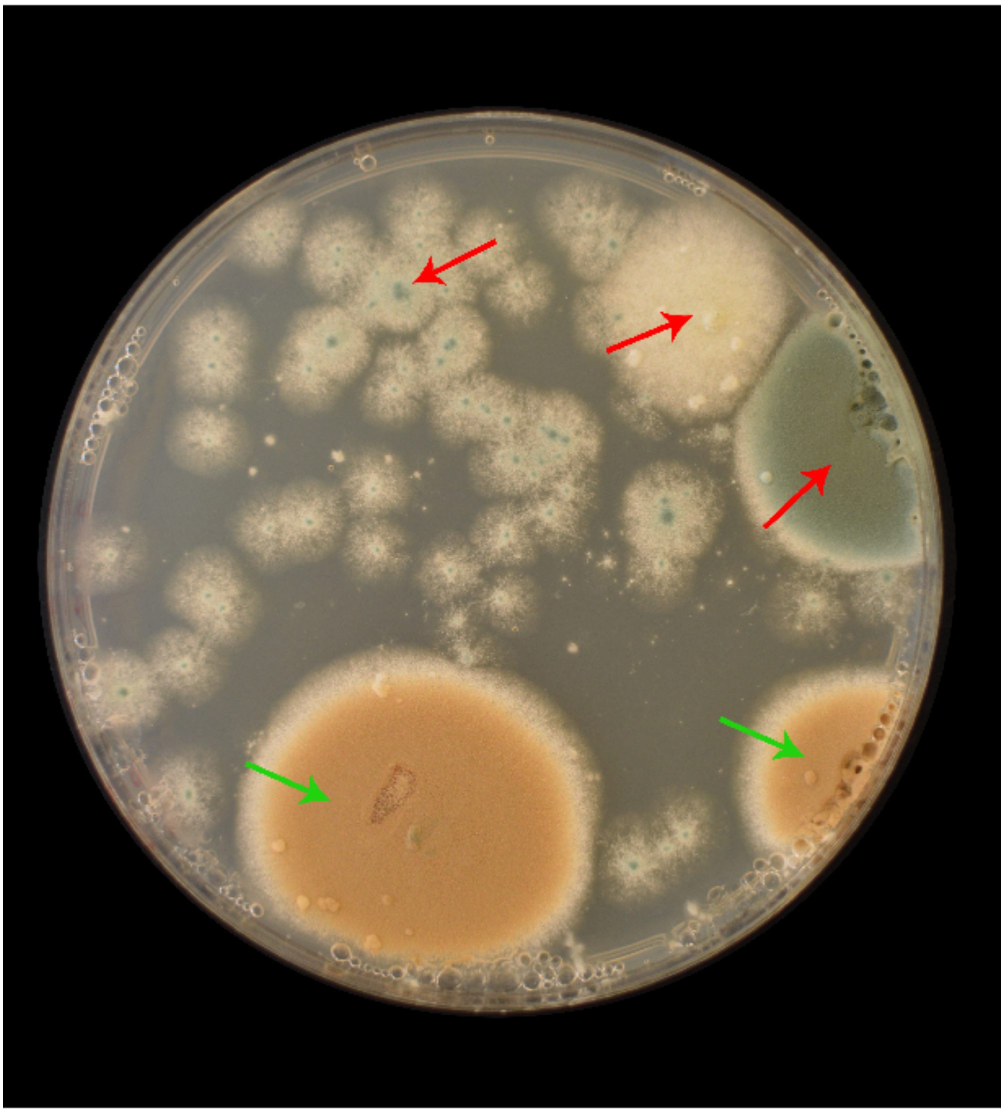

Supplementary Figure 14. Selection plate containing hygromycin-B for *Penicillium roqueforti* transformations. Image shows *Δarp2* successful deletion (green arrows) along with false positive colonies (red arrows).
